# New Eocene Coleoid (Cephalopoda) Diversity from Statolith Remains: Taxonomic Assignation, Fossil Record Analysis, and New Data for Calibrating Molecular Phylogenies

**DOI:** 10.1371/journal.pone.0154062

**Published:** 2016-05-18

**Authors:** Pascal Neige, Hervé Lapierre, Didier Merle

**Affiliations:** 1 Univ. Bourgogne Franche-Comté, CNRS, Biogéosciences, 6 bd Gabriel, 21000, Dijon, France; 2 Département Histoire de la Terre, (MNHN, CNRS, UPMC-Paris6), Paris, France; 3 Département Histoire de la Terre, Sorbonne Universités (CR2P—MNHN, CNRS, UPMC-Paris6), Paris, France; Naturhistoriska riksmuseet, SWEDEN

## Abstract

New coleoid cephalopods are described from statolith remains from the Middle Eocene (Middle Lutetian) of the Paris Basin. Fifteen fossil statoliths are identified and assigned to the Sepiidae (*Sepia boletzkyi* sp. nov.,? *Sepia pira* sp. nov.), Loliginidae (*Loligo clarkei* sp. nov.), and Ommastrephidae (genus indet.) families. The sediments containing these fossils indicate permanent aquatic settings in the infralittoral domain. These sediments range in age from 46 Mya to 43 Mya. Analysis of the fossil record of statoliths (from findings described here, together with a review of previously published data) indicates marked biases in our knowledge. Fossil statoliths are known from as far back as the Early Jurassic (199.3 to 190.8 Mya) but surprisingly, to the best of our knowledge, no record occurs in the Cretaceous. This is a “knowledge bias” and clearly calls for further studies. Finally, we attempt to compare findings described here with fossils previously used to constrain divergence and/or diversification ages of some coleoid subclades in molecular phylogenies. This comparison clearly indicates that the new records detailed here will challenge some estimated divergence times of coleoid cephalopod subclades.

## Introduction

Cephalopods are known from the fossil record as far back as the Cambrian, some 530 million years ago [1]. They span a long history encompassing several severe extinction events. They are characterized by their ability to expand rapidly in terms of diversity in so-called evolutionary radiation events, as shown on present and past examples [2,3]. They are well known in the fossil record because some taxa have easily fossilized external shells. Two major groups continue to populate the marine environment today. The Nautilida, known from the Devonian, more or less 400 million years ago, are now represented by a small number of species belonging to the two genera *Nautilus* and *Allonautilus*. The anatomical organization of the shell of these species is very similar to that acquired when the group first originated, hundreds of millions of years ago. The other group, the coleoids (Coleoidea), is largely diversified with octopods, squids, cuttlefish, and affiliates. The earliest known coleoids are from the Early Carboniferous as attested by the first record of internalization of the shell [1]. This feature is barely observable directly on fossils, but may be identified from shell layers added to the outer surface of the primary shell [1]. Some in particular bear a well-developed internal shell, belemnites, cuttlefish, or spirulids, whereas in others the shell was reduced in size or even completely lost: octopuses, squids or sepiolids. Most coleoids are fast moving animals thanks to their fins and propulsion apparatus, although *Spirula*–the horn squid–is one of several exceptions. There is no doubt today that cephalopods as a whole constitute a monophyletic group [1,4] as demonstrated by molecular, morphological, and anatomical arguments. For example, they all have a centralized and well-developed nervous system, eyes and a visual system with optic lobes, and muscular arms.

The last major mass extinction event at the end of the Cretaceous dramatically affected the cephalopods. Some, including the emblematic ammonites, died out completely. Nautiloidea survived. It is unclear, though, how hard this event hit coleoids. Belemnites disappeared. Other groups, such as Sepiidae: cuttlefish and affiliates, are rare in the fossil record at the end of the Cretaceous but more common after the mass extinction event [5, 6]. However, this pattern of differential extinction/survival is still subject to debate, particularly in the case of coleoids, for at least two reasons. One relates to the Cenozoic cephalopod fossil record. Because most of them did not have a well-developed shell at those times, their fossil record is far from robust. Even for species with a shell, this structure is very fragile and frequently only partially preserved in the fossil record. The second reason, which is obviously linked to the previous one, is that their phylogeny is also unclear, at least when one wants to aggregate present and past species. Things are different when working on present-day groups only, where phylogenetic reconstructions seem to be more clearly established when based on diverse molecular approaches ([7,8] for a review), even if some parts of the phylogeny remain problematic. Moreover, it is worth saying that these are only early days for temporally calibrated phylogenies [7].

In this context, findings of fossil coleoids from the Cenozoic are of particular importance: first they may illustrate life forms unknown today [5] and allow the evolutionary pattern throughout fossil times to be studied; and second they could help in reconstructing and calibrating phylogenies, provided that taxa can be reasonably affiliated to coleoid subclades. However, and as previously claimed, things are difficult because of the poor fossil record of coleoids because they have no well-developed shell, if any. Apart from the rare findings of shell remains, two different and additional strategies may be used to enhance knowledge of fossil coleoids. Soft-part remains of coleoids can be studied in Lagersttäten. These are exceptional outcrops where fossilization conditions allowed organic matter to be mineralized and thus observed (see for example [9]). The other possibility is to focus on other hard, mineralized, structures of coleoids ([10] for a review). Among them are statoliths. They are generally rare in the fossil record. However, some very informative data exist as illustrated by the numerous publications of Malcom R. Clarke, the leading biologist in this field (e.g. see [11–17]), and see S1 Table for an exhaustive presentation of known fossil statoliths.

**Fig 1:**
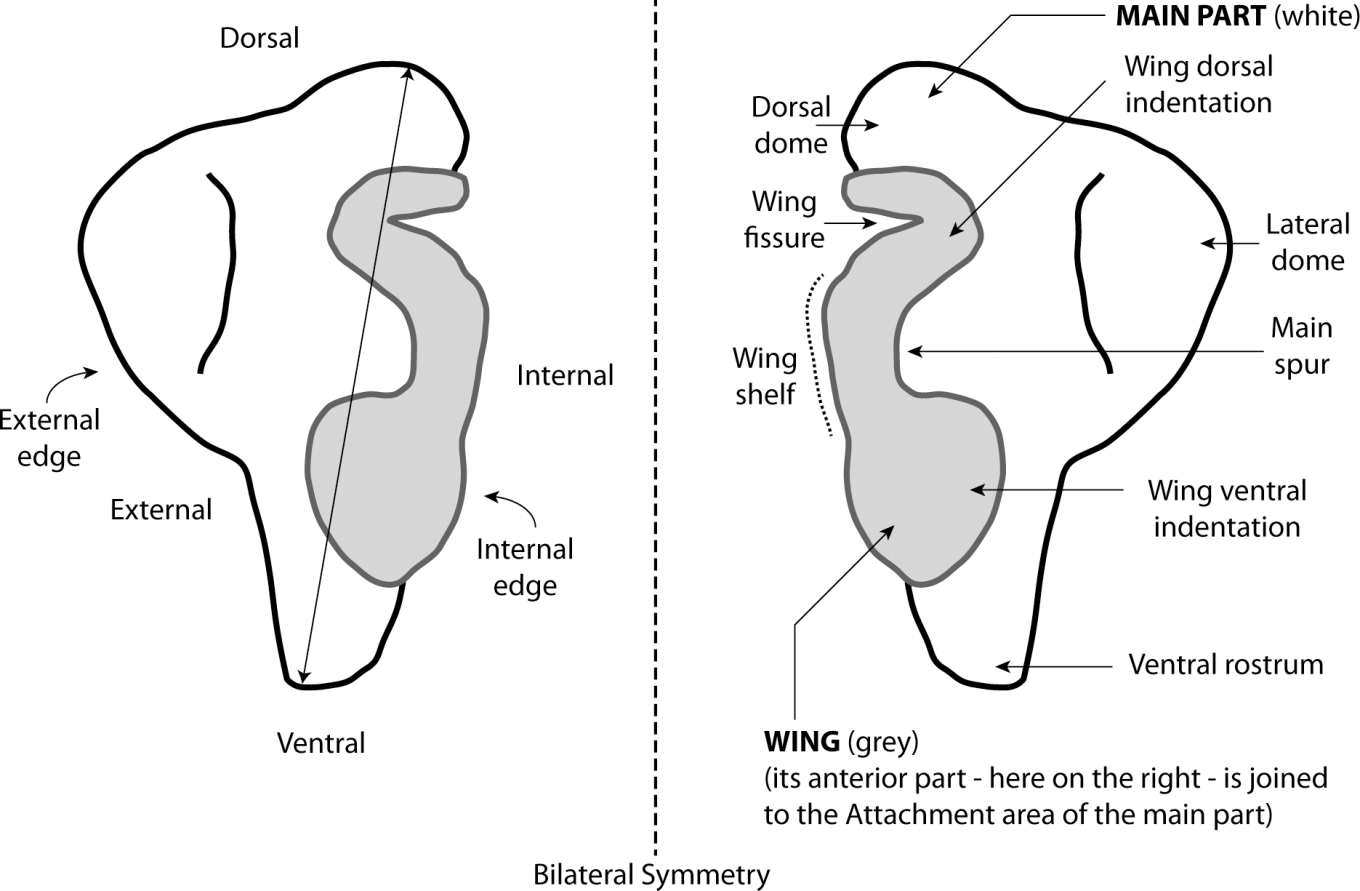
Coleoid statoliths observed in anterior view. The figure shows both the “main part” and the “wing” (which is not observable in posterior view). Left: basic orientation of the right statolith. Arrow indicates standard statolith length measurement. Right: main anatomical parts of the (left) statolith.

Statoliths are aragonitic pieces supported by a set of sensory epithelium cells called crista in fluid-filled cavities called statocysts [18]. Two statocysts, one on the left part, the other on the right, occur in the cartilaginous skull of all coleoid species [17]. Nautiloids have tiny statoliths named statoconia [19,20]. They are ovoid and about 10 μm in size [21]. As far as we know, no fossil statoconica have yet been reported. Statoliths range in size from 0.1 mm up to 3.5 mm for the largest [17]. They are involved in gravity detection along the lengthwise axis of the animal. More recently, it has been suggested that the statolith / statocyst apparatus could be “a detector of multidimensional movement”, including body movements [18]. This has been demonstrated for squids only. Octopods have very distinctive, limpet-like, statoliths. Those from squids, cuttlefish, and affiliates are more complex in shape (Fig 1), with a “main part” displaying more or less protuberant subparts, dorsal and lateral domes, ventral rostrum [12,22]. The second part, the so-called “wing”, is partly fused to the “main part” by the “attachment area”. The “wing” itself contains several anatomical elements: spurs, indentations, fissure and shelf.

At present, little is known about fossil statoliths. Some authors refer to such fossils as “neglected microfossils” [23], suggesting that they may be understudied rather than genuinely rare in the fossil record. Malcolm R. Clarke published many papers which deliberately mixed fossil and recent data (see S1 Table for a complete overview). He also used morphometry abundantly to compare present and past species. Although useful and also applicable to modern statoliths [22,24] this was not possible here because specimens were often incompletely preserved. More recently, Malcolm B. Hart and collaborators [23,25] have published data about fossil statoliths, making explicit reference to the continuation of Clarke’s work. This paucity of studies means that the fossil record of statoliths is poorly documented. However, we believe that the publication of these fossil data is necessary for at least two reasons. First, this could further our understanding of coleoid evolution over time by adding new data to their fossil record. Second, knowledge of statoliths can only increase if micropaleontologists recognize and publish them.

Here, we present new data about statoliths from the Middle Lutetian stage (Middle Eocene) of the Paris Basin. We hope this work will encourage paleontologists to be more attentive to these microfossils. Fossil statoliths recorded here are then discussed in terms of taxonomic affinities mostly using anatomic analysis, and are situated within a broader framework of the history of certain coleoid subclades. Ages for these fossils are given, and results discussed, in order to help with future calibrated phylogenetic reconstructions.

## Geological Settings

**Fig 2:**
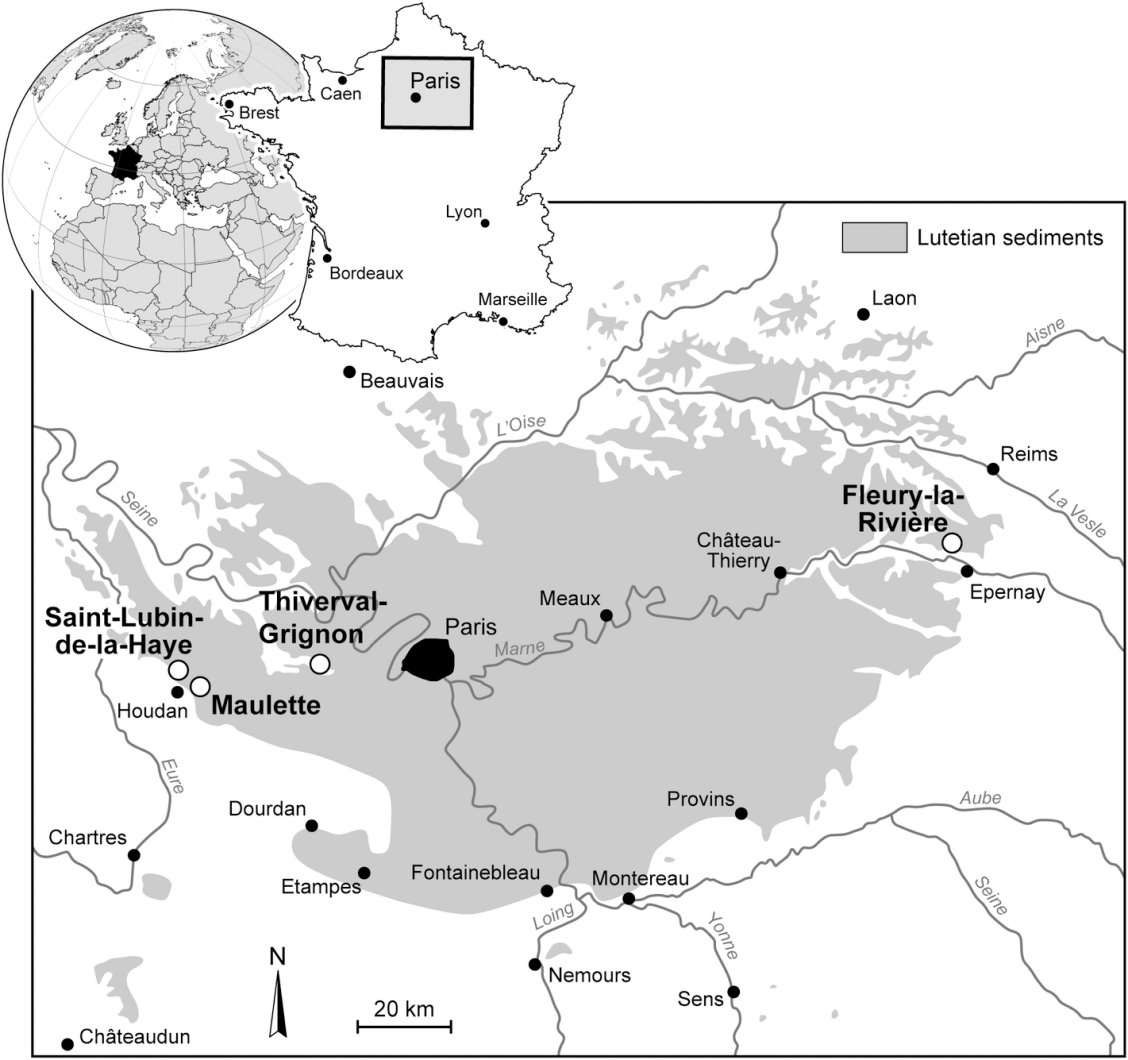
Geographical location of the four Lutetian localities (Thiverval-Grignon, Saint-Lubin-de-la-Haye, Maulette, and Fleury-la-Rivière) yielding statoliths. The localities are indicated on the map of the extension of the Lutetian sediments (from [26] modified).

The statolith material was collected from four sections all dating from the Middle Lutetian of France: Thiverval-Grignon (Yvelines), Maulette (Yvelines), Saint-Lubin-de-la-Haye (Eure et Loir), and Fleury-la-Rivière (Marne). The first three sections are located on the southern margin of the Paris Basin [26] and the fourth on its eastern margin (Fig 2). The owners of the land for Saint-Lubin-de-la-Haye, Maulette, and Fleury-la-Rivière gave permission to conduct the study. Permission for the Thiverval-Grignon site was given by one of us (DM) who is in charge of scientific work at the quarry. Most of the specimens under study were collected by one of us (HL). Field studies concern fossil specimens only, and did not involve endangered or protected species.

**Fig 3:**
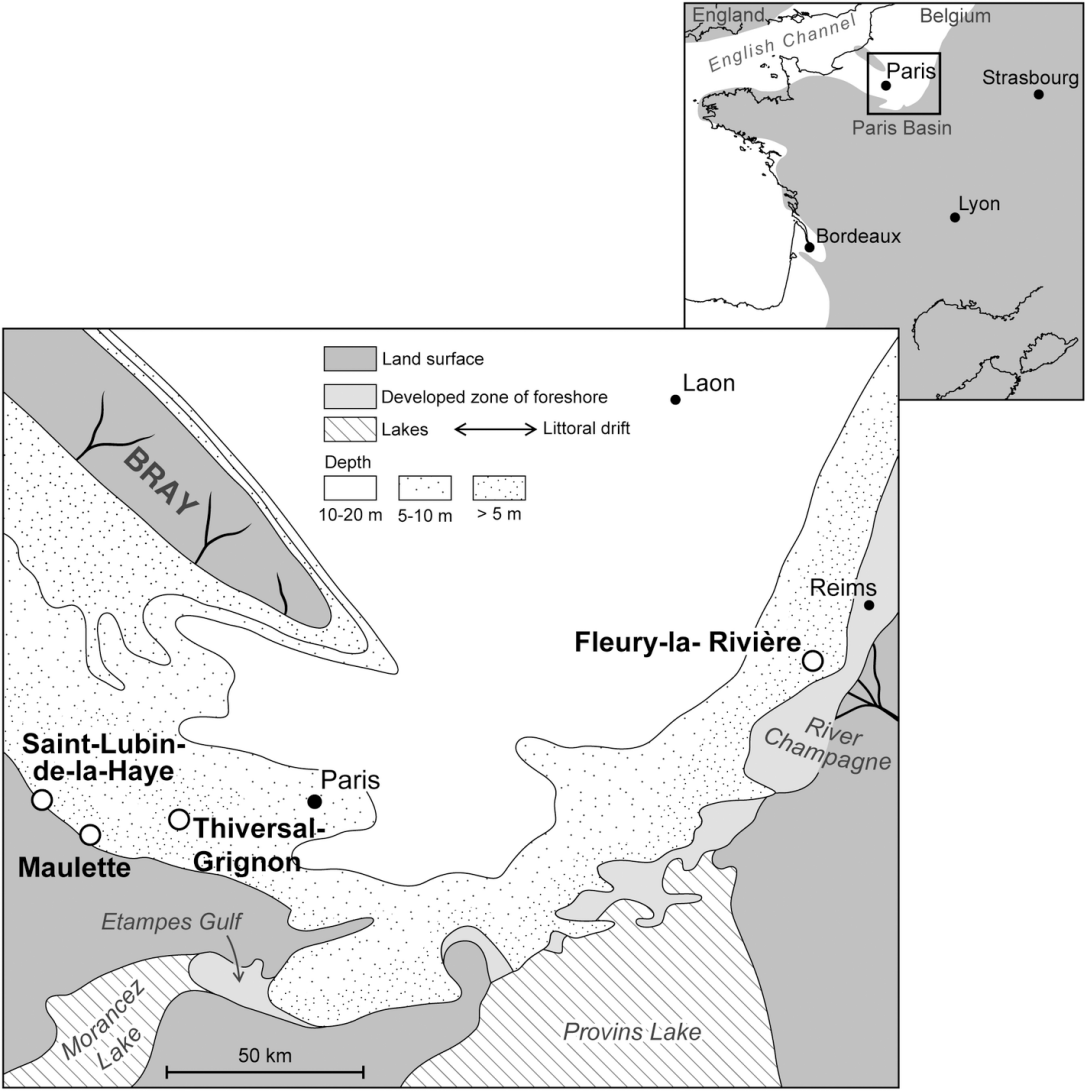
Regional paleogeographical reconstruction for the Middle Lutetian. Location of the four localities yielding statoliths (map from [30], modified).

The Paris Basin is an intracratonic basin, with reduced tectonic activity during the Middle Eocene [27–29]. During the Lutetian, the deposition of a vast carbonate platform with various biofacies [26,30], and the paleogeographic location on the edge of the Atlantic Ocean and northern domains (Fig 3) and close to the Tethys Ocean, provided favorable global conditions for the preservation of a hotspot of paleobiodiversity containing around 3000 species of marine life [31]. The Middle Lutetian displays sediments observed on the southern and the eastern edges of the basin (Fig 2), consisting mostly of non-indurated calcareous sands [30].

### The Thiverval-Grignon section (S1 Fig)

Several great naturalists such as Lamarck, Cuvier, and Brongniart studied the site of “La Falunière” of Thiverval-Grignon which was famed for its remarkable fossil richness. Abrard [32] and Le Calvez and Le Renard [33] gave a composite section, corresponding to different exposures found in the park of Thiverval-Grignon, but not a detailed section of “La Falunière”. This was done later [34–36]. The section given by [36] is the most detailed at the present day and can be used to precisely place the statolith material in its stratigraphical context. The material studied here was collected from two beds: bed 4a (1.0 m thick) is a slightly glauconitic, calcareous sand with *Orbitolites complanatus* Lamarck, 1801 and some miliolids; and immediately above it, bed 3b (0.6 m thick) is a locally lithified clayey limestone also with *O*. *complanatus*, with sea grass [37] of the genus *Cymodoceites*, and with several rasping polyplacophora species. According to the high-resolution sequence stratigraphy of the Lutetian deposits from the Paris Basin [38], these beds belong to parasequence A8 [36].

### The Lubin-de-la-Haye section (S2 Fig)

This section has been known since Abrard [32] who distinguished three beds from the base upward: (1) calcareous red sand with *Ampullina depressa* (Lamarck, 1804) (undefined thickness), (2) glauconitic calcareous and very fossiliferous sand containing many potamidids and batillariids (1.5 m thick), and (3) greenish siliceous limestone with *O*. *complanatus* (7.0 m thick). The material studied here was collected from the boundary between the second and third beds. This locality is not discussed in [38], but it is considered that the second and third beds belong to parasequence A8 (J.-P. Gély, written communication).

### The Maulette section (S3 Fig)

The neighborhood of Maulette contains two paleontological sites the better known of which is “La Tranchée de Maulette” [32,39]. Later [40] studied this locality and supplemented the previous description of the section with new data. Unfortunately, this old section is now inaccessible. However, a temporary site has been recently discovered in the foundations of a house under construction close to the historical site [41]. The section exposes four main beds: from the base, (1) Campanian chalk (undefined thickness), (2) glauconitic calcareous sand with many branchy pebbles and shells (0.4 m thick), (3) very fossiliferous red calcareous sand with *Cubitostrea elegans* (Deshayes, 1832) (2.0 m thick), and (4) poorly fossiliferous whitish sand with *Granulobium* (0.3 m thick). The statoliths from this new site were collected for the base of the third bed. A bed containing numerous *C*. *elegans* is not reported in the section of “La Tranchée de Maulette” described by [40]. However, it is reported in the Houdan section [32,40] and corresponds to parasequence A7 (J.-P. Gély, written communication).

### The Fleury-la-Rivière section (S4 Fig)

This paleontological site, also called “La Cave aux Coquillages”, is close to the classical site of Damery for which the section and its place in the sequence stratigraphy of the Lutetian deposits from the Paris Basin has been previously published [38]. The section of “La Cave aux Coquillages” is given by [34]. The statolith material was collected from immediately above the *Campanile giganteum* (Lamarck, 1804) bed in a dark clayey sand with many *Sigmesalia* (0.5 m thick). This bed (bed 7) can be correlated to parasequence A8, starting immediately above the *C*. *giganteum* bed, corresponding to parasequence A7 [38].

## Age and Paleoenvironment of the Sediments Containing Statoliths

The statolith material collected from the sections is from parasequence A8, with the exception of the La Tranchée de la Maulette section, which belongs to parasequence A7. No radiometric dating for these parasequences currently exists, but some can be ascribed to the Lutetian [42]. However, parasequences A7-A8 belong to the biozone NP15 [30,43] and thus an approximate age of 46 to 43 Mya can be attributed [44].

The exceptionally rich mollusk assemblages at Thiverval-Grignon are structured by a general shallowing of their environments. By comparison with parasequence A7, a significant facies change is observed within parasequence A8, with calcareous bioclastic sand containing *Orbitolites*, miliolids, and a highly diverse fauna of mollusks, indicating a probable reduction of paleodepth to less than 20 m. Within parasequence A8, beds 4a (S1 Fig) and 3b correspond to a low-energy, inner-platform depositional environment. Paleoclimatic reconstructions of these two beds from mollusks support subtropical regional conditions [35,45] with temperatures of the warmer months of c. 28–30°C [35]. The assemblage of bed 4a is dominated by the phytophagous gastropods *Cirsochilus* and *Bittium* associated with *Ptychocerithium*, *Collonia*, *Phasianella*, *Tectus*, and rissoids. The infauna is dominated by suspension feeders, such as *Haustator* and different species of *Pitar*. Numerous remains of reworked *Seraphs sopitus* (Solander in Brander, 1766) have been collected from this bed, but the shells are so broken that it was impossible to count the specimens. The biodiversity of the assemblage of bed 3b is poorer than that of bed 4a. It is dominated by the phytophagous gastropod genus *Ptychocerithium* associated with *Angaria*, *Cirsochilus*, *Bittium*, *Collonia*, *Tectus*, and rissoids. Several chiton species represented by numerous shell plates complete this phytophagous assemblage. The infauna is dominated by suspension feeders such as *Sigmesalia favrei* (Le Renard, 1994) and different species of carditids. Although a faunal turnover can be observed from beds 4a to 3b, the dominance of the epifauna indicates an environment rich in sea grass attested by the presence of algae [46] or *Cymodoceites* [47].

The Saint-Lubin-de-la-Haye and La Tranchée de la Maulette outcrops are the most westerly of the Middle Lutetian and contain every indication that the coastline was close. Numerous mollusks have been reported from the base of the limestone with *O*. *complanatus* at Saint-Lubin-de-la Haye (S2 Fig) [32]: *Anomia anomialis* (Lamarck, 1819) associated with bryozoans, *Parvilucina albella* (Lamarck, 1806), *Cardita* sp, *Loxocardium bouei* (Deshayes, 1858), *Callista elegans* (Lamarck, 1806), *Sigmesalia fasciata* (Lamarck, 1804), *Serratocerithium denticulatum* (Lamarck, 1806), and some corals *Turbinolia sulcata* Lamarck, 1816. This assemblage together with stenohaline organisms is indicative of full marine conditions. Numerous stenohaline mollusks have also been collected from the bed with *C*. *elegans* at La Tranchée de la Maulette (S3 Fig) including large mollusks such as *Volutocorbis crenulifera* (Bayan, 1870), *Clavilithes noae* (Holten, 1802), *Volutilithes muricinus* (Lamarck, 1802), *Chama subgigas* d’Orbigny, 1850, *Venericor planicosta* (Lamarck, 1801), and *Crassatella gibbosula* Lamarck, 1805 [41].

As at Thiverval-Grignon, the Fleury-la-Rivière section shows a transition from parasequence A7 (bed 6) to parasequence A8 (bed 7) characterized by a shallowing of the environments (S4 Fig). Bed 6 contains numerous reworked *Campanile giganteum*, a species that almost disappears in bed 7. The base of bed 7 is very rich in shell fragments, whereas large shells become uncommon. The great abundance of fragments of green algae as well as the sedimentary figures suggest tidal influences. However, this facies remained under permanent aquatic conditions in the infralittoral domain [34]. The great prevalence of the endobenthic suspension feeder *Sigmesalia intermedia* (Deshayes, 1832) is the most remarkable biological element and indicates a muddy bottom.

## Material

Most of the specimens under study were collected by one of us (HL). The first statoliths were found while searching for fish otoliths. Thereafter, a systematic search was made. Sediment from 50 kg batches was stirred gently in water. Residues between 2 mm and 0.2 mm were retained. These residues were fractionated in water following an elutriation process. Simply speaking, water enters through the side of a tank topped by a tube. The sediment is deposited in the upper part of the tube. Weak particles are pushed out and evacuated near the top of the tube whereas heavy ones are settle in the bottom of the tank. The heavy residues contained the whole statoliths and fish otoliths (sagittae and lapilli). These different matrixes were again fractionated in water using meshes between 2 and 0.2 mm. In these last sieved sediments, otoliths and statoliths of small size were easily identified by their reddish coloration. Fifty Lutetian locations relatively rich in fish in the Paris Basin were checked. Statoliths were found in four of them only. Statoliths vary in number between 0 and 2 per 50 kg batch. A previous study [12] indicated that the presence of statoliths is associated with locations or beds in which fish otoliths (sagittae) are concentrated. In the Lutetian stage, from our results, mainly in marly lime beds, the presence of statoliths is associated with fish lapilli rather than sagittae. Unfortunately Lutetian lapilli have not yet been described. Most of the sagittae found in these statolith locations belong to distinct fish species. However some are common members of suborder Clupeoidei (*Chirocentrus exilis*, “*Clupeidea” schultzi*, *Pellona* sp.).

A total of 26 statolith specimens were isolated. Fifteen were sufficiently well preserved to be studied and identified. The others were poorly preserved, precluding any systematic attribution. Table 1 gives details of the specimens studied. All the fossil specimens used here are housed in the paleontology collection (MNHN.F) at the Muséum national d’Histoire naturelle (Paris, France). These specimens were compared to several statoliths of present and past cephalopod species. S1 Table gives an exhaustive list of known fossil statoliths from the literature, with indications of specimens published several times. Comparisons with recent species were based in part on literature analysis, but were mostly made with specimens collected by one of us (PN) during the Cephalopod Parasite Workshop at Banyuls-sur-Mer (November 1996) organized by Sigurd von Boletzky (see [22,48] for detailed studies on statoliths and beak morphometry, respectively). This collection is housed at the University of Burgundy (Dijon, France) abbreviated as (UBGD). Some of the statoliths from this collection are figured here for the first time. In order to connect the present publication with the initial one dealing with morphometry [22], specimen numbers of the morphometric procedure are recalled here after their new official collection number. Specimens were photographed using Scanning Electron Microscopy (SEM Hitachi TM-1000 at the Biogeosciences laboratory, Dijon, France).

**Table 1:** Details of fossil statoliths studied here.

Locality	Specimen Number	Taxa	Remark	Figuration in the present paper
Thiverval-Grignon	MNHN.F.A53747	*Loligo clarkei* sp. nov.	Paratype	Fig 7A3
Thiverval-Grignon	MNHN.F.A53748	*Loligo clarkei* sp. nov.	Paratype	Fig 7A4
Thiverval-Grignon	MNHN.F.A53749	*Loligo clarkei* sp. nov.	Paratype	not figured
Thiverval-Grignon	MNHN.F.A53750	*Loligo clarkei* sp. nov.	Paratype	not figured
Thiverval-Grignon	MNHN.F.A53751	*Loligo clarkei* sp. nov.	Paratype	not figured
Thiverval-Grignon	MNHN.F.A53758	Ommastrephidae, genus indet.	1 specimen	Fig 8A1
Thiverval-Grignon	MNHN.F.A53759	Ommastrephidae, genus indet.	1 specimen	Fig 8A2
Thiverval-Grignon	MNHN.F.A53753	*Sepia boletzkyi* sp. nov.	Holotype	Fig 4A1
Thiverval-Grignon	MNHN.F.A53754	*Sepia boletzkyi* sp. nov.	Paratype	Fig 4A2
Thiverval-Grignon	MNHN.F.A53755	*Sepia boletzkyi* sp. nov.	Paratype	Fig 4A3
Thiverval-Grignon	MNHN.F.A53756	*Sepia boletzkyi* sp. nov.	Paratype	Fig 4A4
Thiverval-Grignon	MNHN.F.A53757	?*Sepia pira* sp. nov.	Holotype	Fig 4B
Thiverval-Grignon	–	No taxonomic identification	6 specimens	not figured
Fleury-la-Rivière	MNHN.F.A53745	*Loligo clarkei* sp. nov.	Holotype	Fig 7A1
Fleury-la-Rivière	MNHN.F.A53746	*Loligo clarkei* sp. nov.	Paratype	Fig 7A2
Fleury-la-Rivière	–	No taxonomic identification	2 specimens	not figured
Maulette	–	No taxonomic identification	3 specimens	not figured
Lubin-de-la-Haye	MNHN.F.A53752	*Loligo clarkei* sp. nov.	Paratype	Fig 7A5

### Nomenclatural Acts

The electronic edition of this article conforms to the requirements of the amended International Code of Zoological Nomenclature, and hence the new names contained herein are available under that Code from the electronic edition of this article. This published work and the nomenclatural acts it contains have been registered in ZooBank, the online registration system for the ICZN. The ZooBank LSIDs (Life Science Identifiers) can be resolved and the associated information viewed through any standard web browser by appending the LSID to the prefix “http://zoobank.org/”. The LSID for this publication is: urn:lsid:zoobank.org:pub: 90759BF6-D847-4F57-88AE-A59D99721BB4. The electronic edition of this work was published in a journal with an ISSN, and has been archived and is available from the following digital repositories: PubMed Central, LOCKSS. Specimens studied can be found in the following repositories: (1) Muséum national d’Histoire naturelle, Paris, collection de Paléontologie (France): MNHN.F.A53745, MNHN.F.A53746, MNHN.F.A53747, MNHN.F.A53748, MNHN.F.A53749, MNHN.F.A53750, MNHN.F.A53751, MNHN.F.A53752, MNHN.F.A53753, MNHN.F.A53754, MNHN.F.A53755, MNHN.F.A53756, MNHN.F.A53757, MNHN.F.A53758, MNHN.F.A53759, and (2) Université de Bourgogne, Dijon (France): UBGD 30128 SD, UBGD 30044 SD, UBGD 30045 SG, UBGD 30051 SD, UBGD 30104 SG, UBGD 30155 SD.

## Systematic Paleontology

Class Cephalopoda Cuvier, 1797

Subclass Coleoidea Bather, 1888

Superorder Decabrachia Haeckel, 1866, (see [49])

Order Sepiida Zittel, 1895

Family Sepiidae Keferstein, 1866

**Genus *Sepia* Linnaeus, 1758**

Type species.- *Sepia officinalis* Linnaeus, 1758 by Linnaean tautonymy.

***Sepia boletzkyi* sp. nov.**

urn:lsid:zoobank.org:act: CF26E545-1604-4351-968B-18D6F6E2EDFE

Figs 4A1–4A4 and 5A

**Fig 4:**
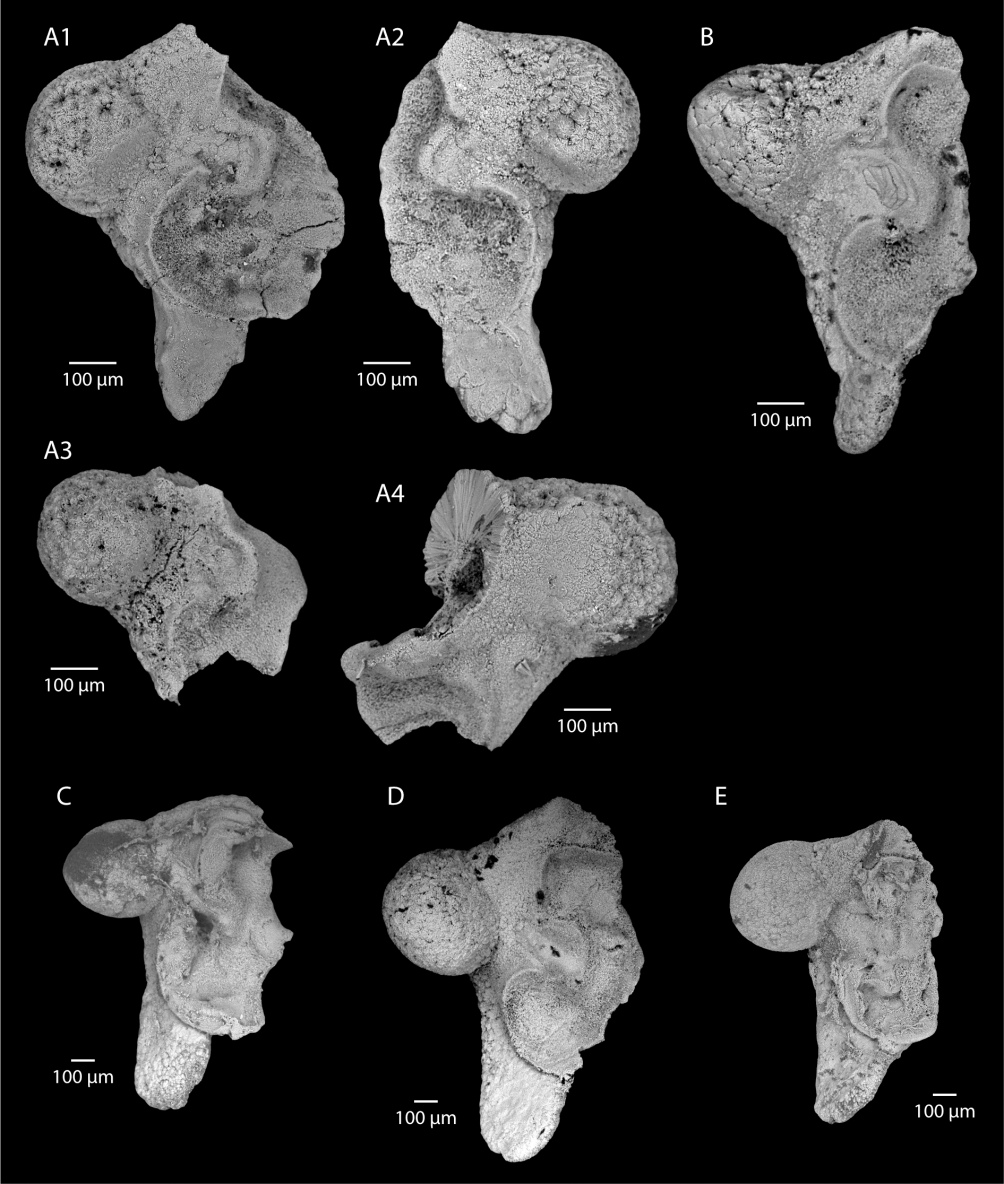
Eocene (A-B) and Recent (C-E) statoliths of *Sepia*. A. *Sepia boleztkyi* sp. nov. (Lutetian, Paris Basin), A1: holotype, view of anterior side, right statolith (MNHN.F.A53753), A2: paratype, view of anterior side, left statolith (MNHN.F.A53754). A3: view of anterior side, right statolith, partially broken (MNHN.F.A53755). A4: paratype, view of anterior side, left statolith, partially broken (MNHN.F.A53756). B.? *Sepia pira* sp. nov. (Lutetian, Paris Basin), holotype, view of anterior side, right statolith (MNHN.F.A53757). C. *Sepia officinalis* Linneaus, 1758, view of anterior side, right statolith (UBGD 30044 SD (CDN044 in [22]), immature male specimen from the western Mediterranean Sea (area of Banyuls-sur-Mer), mantle length: 67.66 mm. D. *Sepia orbignyana* Férussac, 1826, view of anterior side, right statolith (UBGD 30128 SD (CDN128 in [22]), mature male specimen from the western Mediterranean Sea (area of Banyuls-sur-Mer), mantle length: 67.59 mm. E. *Sepia elegans* Blainville, 1827, view of anterior side, right statolith (UBGD 30051 SD (CDN051 in [22]), mature female specimen from the western Mediterranean Sea (area of Banyuls-sur-Mer), mantle length: 41.96 mm.

**Fig 5:**
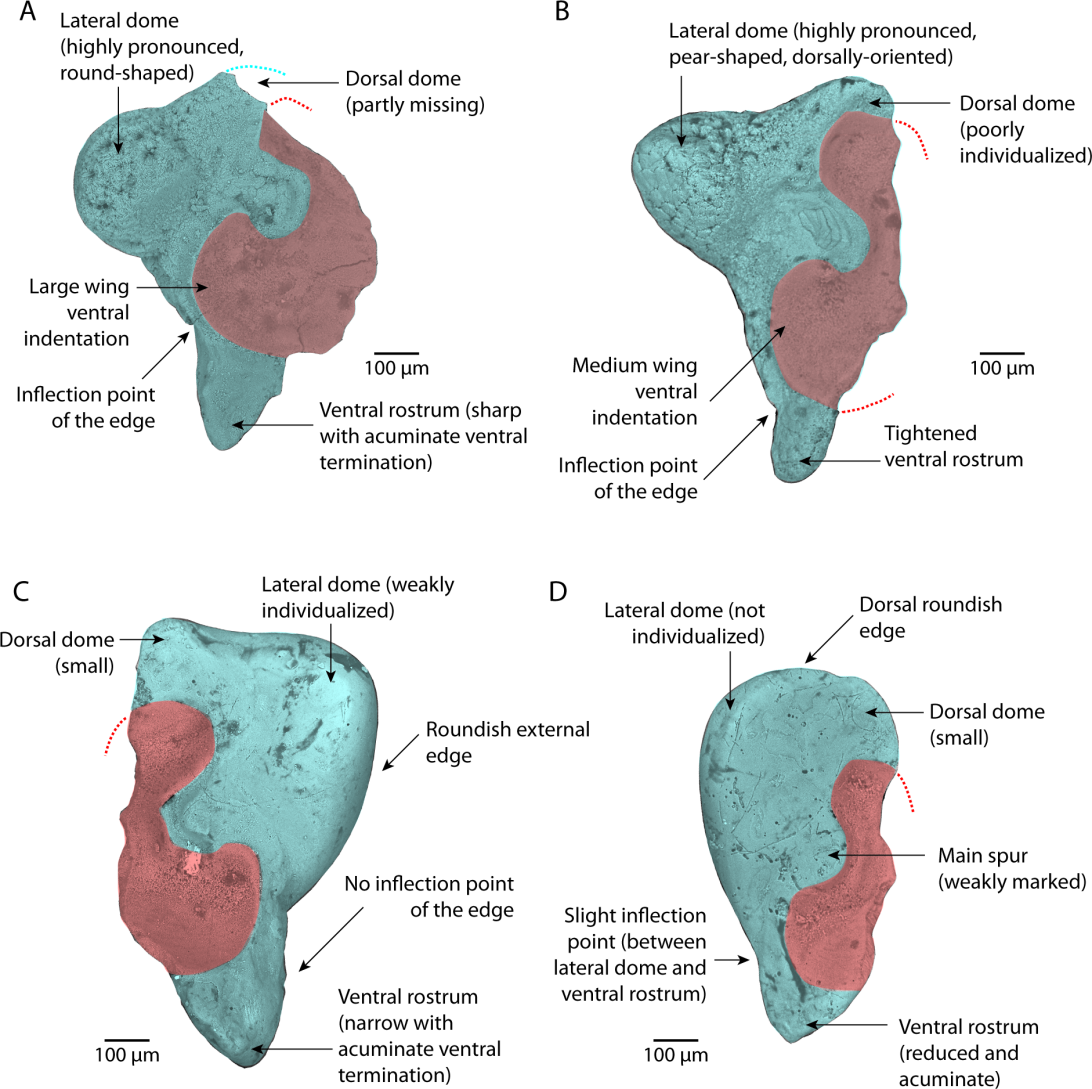
Anatomical overview of Lutetian statoliths. Only main diagnostic features are indicated. A. *Sepia boletzkyi* sp. nov., holotype, view of anterior side, right statolith (MNHN.F.A53753). B.? *Sepia pira* sp. nov., holotype, view of anterior side, right statolith (MNHN.F.A53757). C. *Loligo clarkei* sp. nov., holotype, view of anterior side, left statolith (MNHN.F.A53745). D. Ommastrephidae genus indet., view of anterior side, right statolith (MNHN.F.A53758).

### Derivation of name

Named from Sigurd von Boletzky, leading authority on cephalopods, and especially for his immense contribution to developing close ties among scholars working on present and past cephalopods.

### Type material

Holotype: MNHN.F.A53753 (Thiverval-Grignon, La Falunière, Yvelines, France, Fig 4A1); Paratypes: MNHN.F.A53754 (Thiverval-Grignon, La Falunière, Fig 4A2), MNHN.F.A53755 (Thiverval-Grignon, La Falunière, Fig 4A3), and MNHN.F.A53756 (Thiverval-Grignon, La Falunière, Fig 4A4).

### Type locality

Thiverval-Grignon, La Falunière, Middle Lutetian with an approximate age of 46 to 43 Mya.

### Diagnosis

The introduction of a new species name is justified because (1) the edge between the lateral dome and the ventral rostrum has no inflection point in recent species, and (2) the wing ventral indentation is only slightly larger than the dorsal indentation in recent species

### Description

Statoliths slightly longer than they are wide (dorso-ventral size (see “statolith length” in Fig 1), similar to external-internal size, but internal part of the wing partly missing preventing measurements). Dorsal dome not completely preserved, but poorly individualized. Lateral dome prominent with a characteristic near perfect rounded-shape. Rostrum from half to two-thirds of total height. Ventral rostrum elongate with a more or less sharp and acuminate ventral termination. Edge between lateral dome and ventral rostrum with marked inflection point half way along. Wing not completely preserved on any of the four specimens. Its external part displays a very large ventral indentation almost reaching the statolith’s external edge. Main spur also prominent. Wing dorsal indentation small, and less pronounced than the ventral one.

### Comparison

**Fig 6:**
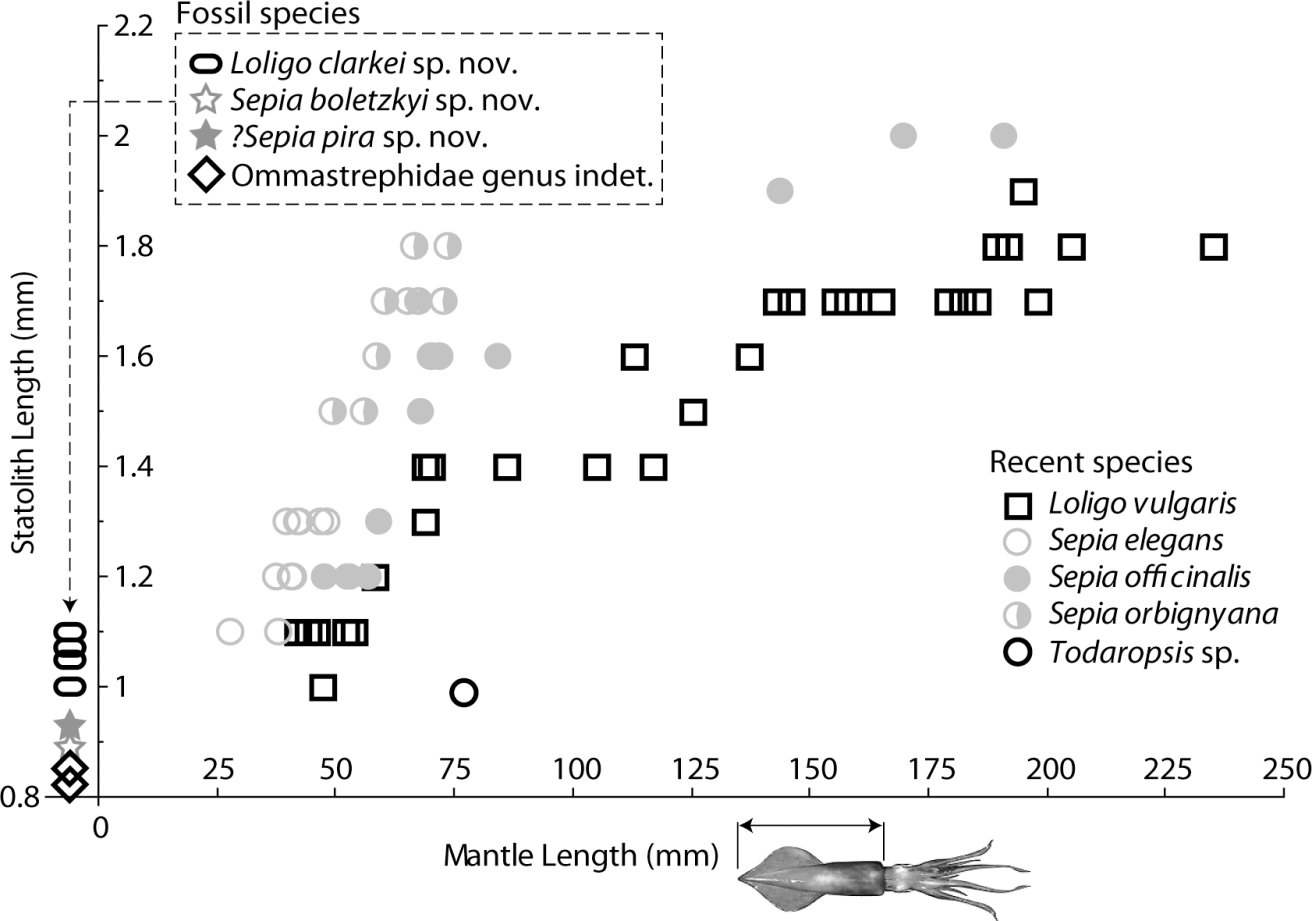
Size of statoliths. Size is given for some recent species and for fossil species under study (see S2 Table), and compared to mantle length (ML) for recent species only. Recent specimens measured here are those used for morphometric analysis (see [22] and text).

The shape and individualization of the lateral dome clearly indicate that this species belongs to the genus *Sepia*. This may be verified by comparison with the statoliths of the three Mediterranean *Sepia* species (see [50] for a description of these species): *S*. *officinalis* (Fig 4C), *Sepia orbignyana* Férussac, 1926 (Fig 4D), and *Sepia elegans* Blainville, 1827 (Fig 4E). This rounded shape seems to be an apomorphic character of the genus. Fossil specimens smaller than recent *Sepia* statoliths (Fig 6, S2 Table), for small mature specimens (Fig 4E), and even for immature specimens of large species (Fig 4C). This seems to indicate that specimens associated with these fossil statoliths were small in size (probably a mantle size less than 40 mm). At the present day, it is not possible to interpret this small size, which could indicate juveniles or small mature specimens.

### Occurrence

Only known from the type locality (see Table 1).

***?Sepia pira* sp. nov.**

urn:lsid:zoobank.org:act: 4EFDE919-3F71-4262-A3D8-54E8E3FB0E81

Figs 4B and 5B

### Derivation of name

Named from the pear-shape of the lateral dome.

### Type material

Holotype: MNHN.F.A53757 (Thiverval-Grignon, La Falunière, Yvelines, France, Fig 4B).

### Type locality

Thiverval-Grignon, La Falunière, Middle Lutetian with an approximate age of 46 to 43 Mya.

### Diagnosis

The shape of the lateral dome is unusual: it is both individualized and pear-shaped. For that reason its affiliation to the genus *Sepia* remains hypothetical (compared to the three Mediterranean species, Fig 4C–4E). This pear-like shape of the lateral dome seems unique and calls for the introduction of a new species name.

### Description

Statolith typically longer than it is wide (dorso-ventral size is 0.93 mm). Dorsal dome moderately individualized. Lateral dome prominent with a characteristic pear-shape and dorsally-oriented. Rostrum nearly half of the total height. Ventral rostrum tightened with a rounded ventral termination. Edge between lateral dome and ventral rostrum has a marked inflection point close to the ventral edge. Wing not completely preserved. Its external part displays a large ventral indentation almost reaching the external edge of the statolith. Main spur also prominent. Wing dorsal indentation slightly less pronounced than the ventral one.

### Comparison

As with the previous species (*Sepia boletzkyi* n. sp.), this new species has an inflection point on the edge between the lateral dome and the ventral rostrum. The shape of the wing resembles that of recent *Sepia* species. This new fossil species is smaller than recent *Sepia* statoliths (Fig 6, S2 Table), indicating, as previously stated, that specimens associated with these fossil statoliths were small in size (probably a mantle size less than 40 mm).

### Occurrence

Only known from the type locality (see Table 1).

Order Teuthida Naef, 1916

Suborder Myopsina Orbigny, 1841

Family Loliginidae Lesueur, 1921

**Genus *Loligo* Lamarck, 1798**

Type species.- *Loligo vulgaris* Lamarck, 1798 by subsequent designation by [51].

***Loligo clarkei* sp. nov.**

urn:lsid:zoobank.org:act: B9E5DC6A-2591-424F-AC21-EFF782E070E2

Figs 7A1–7A5 and 5C

**Fig 7:**
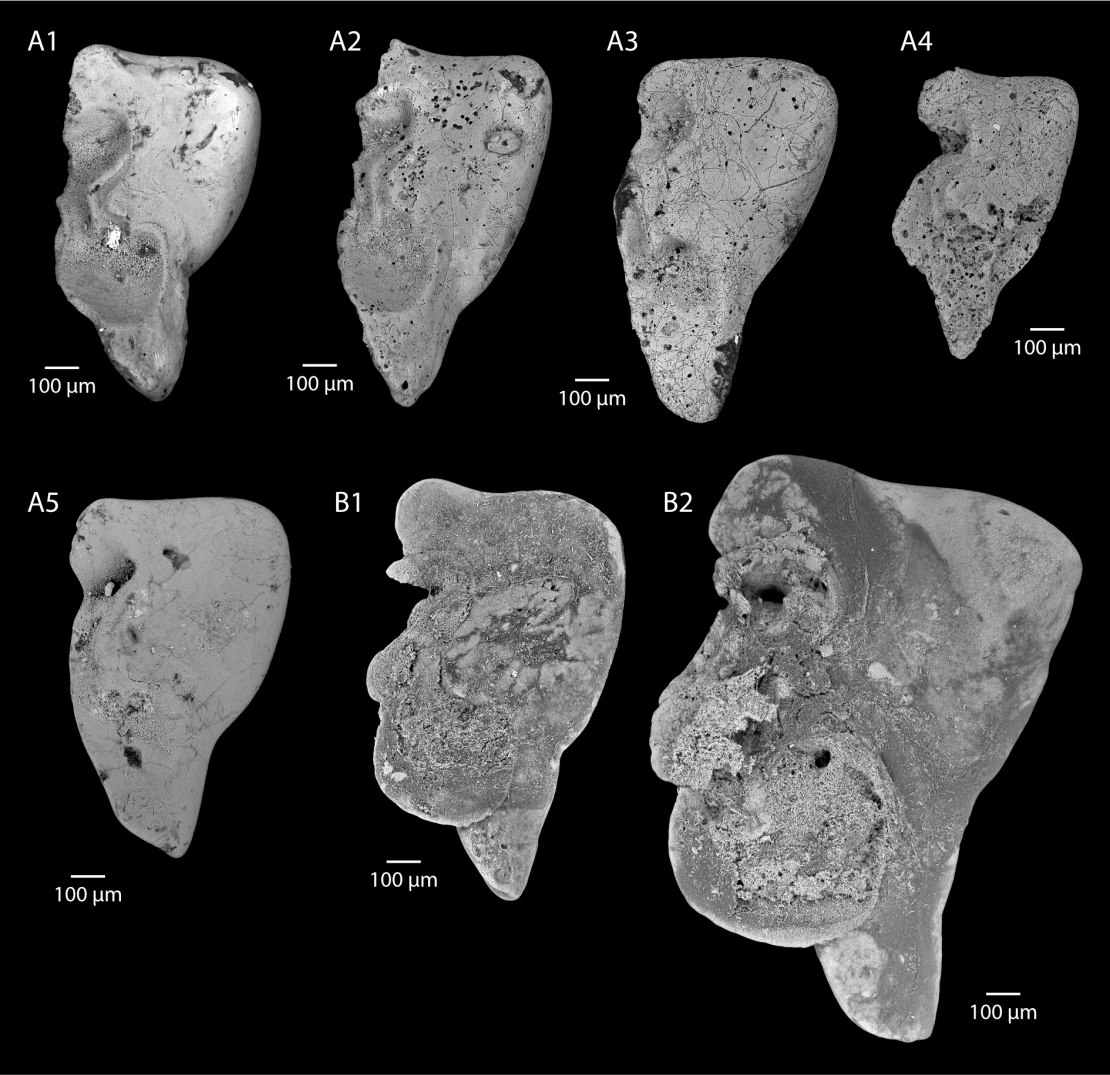
**Eocene (A) and Recent (B) statoliths of *Loligo*.** A1 to A5. *Loligo clarkei* n. sp. (Lutetian, Paris Basin), A1: holotype, view of anterior side, left statolith (MNHN.F.A53745), A2: paratype, view of anterior side, left statolith (MNHN.F.A53746), A3: paratype, view of anterior side, left statolith (MNHN.F.A53747), A4: paratype, view of anterior side, left statolith (MNHN.F.A53748), A5: view of anterior side, left statolith (MNHN.F.A53752). B. *Loligo vulgaris* Lamarck, 1798, B1: view of anterior side, left statolith (UBGD 30045 SG (CDN045 in [22]), immature specimen from the western Mediterranean Sea (area of Banyuls-sur-Mer), mantle length: 70.00 mm, B2: view of anterior side, left statolith (UBGD 30104 SG (CDN104 in [22]), mature male specimen from the western Mediterranean Sea (area of Banyuls-sur-Mer), mantle length: 235.00 mm.

### Derivation of name

Named after Malcom Roy Clarke (1930–2013), leading authority on cephalopods, and a pioneer in the field of fossil statolith studies (see [52,53] for biographies).

### Type material

Holotype (nearly complete): MNHN.F.A53745 (Fleury-la-Rivière, Fig 7A1); Paratypes: MNHN.F.A53746 (Fleury-la-Rivière, Fig 7A2), MNHN.F.A53747 (Thiverval-Grignon, La Falunière, Fig 7A3), MNHN.F.A53748 (Thiverval-Grignon, La Falunière, Fig 7A4), MNHN.F.A53752 (Lubin-de-la-Haye, Fig 7A5), MNHN.F.A53749 (Thiverval-Grignon, La Falunière, not figured), MNHN.F.A53750 (Thiverval-Grignon, La Falunière, not figured), MNHN.F.A53751 (Thiverval-Grignon, La Falunière, not figured).

### Type locality

Fleury-la-Rivière; Middle Lutetian with an approximate age of 46 to 43 Mya.

### Diagnosis

The present species is considered to be similar to the species first described under the name *Loligo* sp. A by [12] of Lutetian age and from the Wallmeyer’s Bluff area (Hanover County, Virginia, USA). The same specimens were figured again by [16]. The absence of any groove or distinctive character between the lateral dome and the center of the main part of the statolith is observed both on Clarke’s specimens and those studied here. The first authors [12] did not give a species name because at that time they had only one small specimen (0.84 mm), suggesting it could be a juvenile. The specimens we add here to that species (8 more) warrant introducing a new species name.

### Description

Statoliths typically longer than wide (dorso-ventral size ranges from 1.00 to 1.10 mm). Dorsal dome small and in the continuation of dorsal area of the main part. Lateral dome narrow at its ventral end and widest near its dorsal end. However, this dome is far from being well individualized: the contact between the dome and the center of the main part of the statolith is difficult to localize as there is no groove. It imparts a characteristic triangular shape to the statolith associating a flat dorsal edge with a roundish external edge. The rostrum extends for two-thirds of the total height. Small ventral rostrum with acuminate ventral termination. Edge between lateral dome and ventral rostrum has no inflection point. Wing not completely preserved. Its external part displays a larger ventral than dorsal indentation, but the difference in size between them remains small. Main spur more or less marked.

### Comparison

The global shape and the lack of well-individualized lateral dome indicate that this species belongs to the genus *Loligo*. The similarity with *L*. *vulgaris* is marked (Fig 6F1 and 6F2). See also [12] for comparisons with *Loligo forbesii* Steenstrup, 1856. *Loligo clarkei* sp. nov is different from the second Lutetian species, *Loligo applegatei* Clarke and Fitch, 1979, which has a diagnostic sharply pointed lateral dome. This new fossil species has statolith sizes from 1.00 to 1.10 mm. Compared with statolith sizes of *L*. *vulgaris* (Fig 6, S2 Table) this seems to indicate a mantle size of 42.0 to 54.0 mm.

### Occurrence

The new species is known from three localities (see Table 1) in the Paris Basin (Thiverval-Grignon, Lubin-de-la-Haye, and Fleury-la-Rivière), of Lutetian age (Middle Eocene), and with an approximate age of 46 to 43 Mya. It is also known from one specimen [12] under the name *Loligo* sp. A, of Lutetian age from the Wallmeyer’s Bluff area (Hanover County, Virginia, USA). This occurrence on the east coast of the USA is congruent with the presence of the species in the Paris Basin, and suggests a probably pelagic mode of life over a large area covering at least the northern part of the Atlantic Ocean. This type of geographical distribution (covering a large oceanic area, from one coast to the other) is very common for some recent squids, which are grouped together as “oceanic squids” [54].

Suborder Oegopsina Orbigny, 1845

Family Ommastrephidae Steenstrup, 1857

**Genus indet.**

Fig 8A1 and 8A2

**Fig 8:**
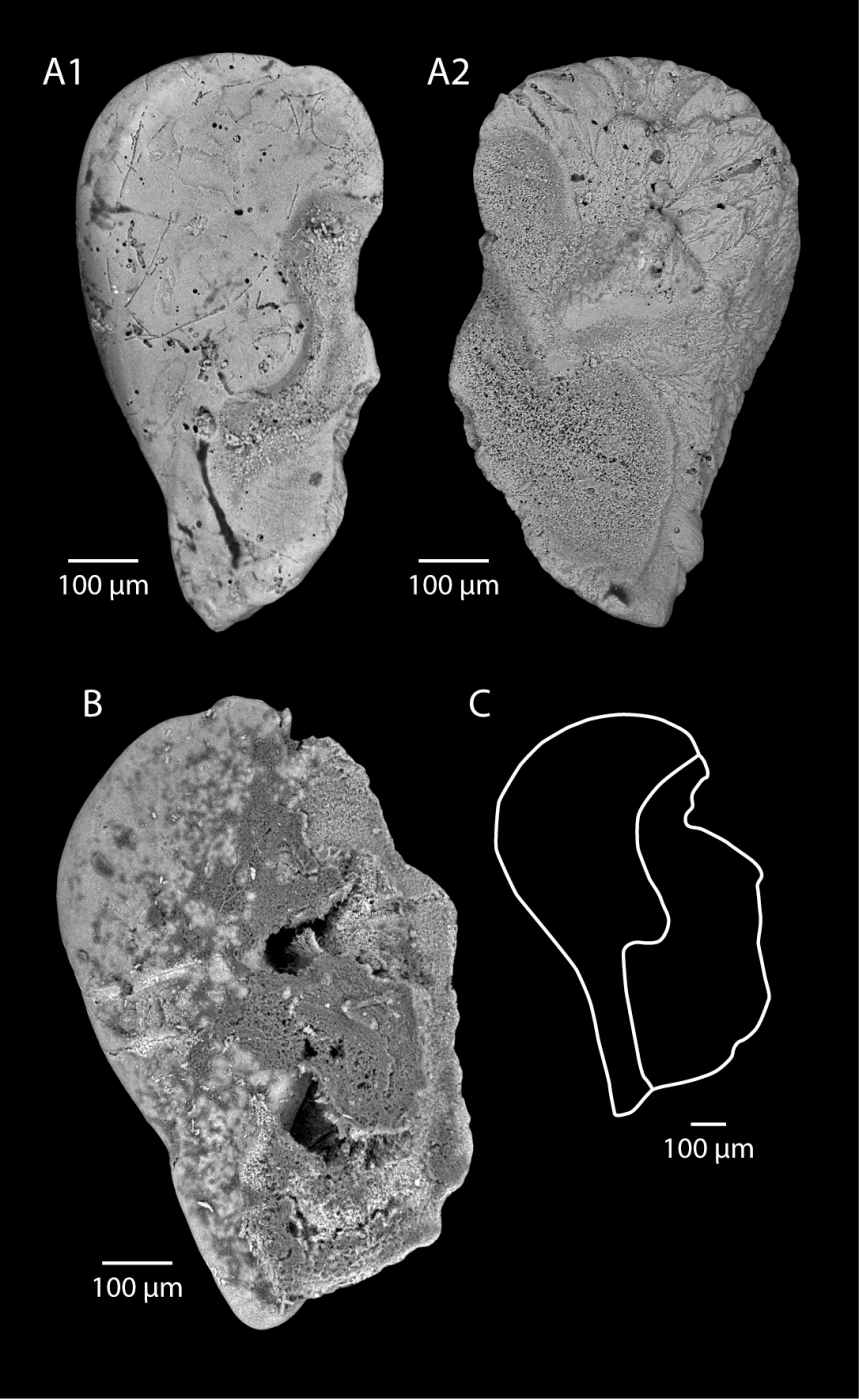
**Eocene (A) and Recent (B-C) statoliths of Ommastrephidae.** A1, A2. Ommastrephidae genus indet. (Lutetian, Paris Basin), A1: view of anterior side, right statolith (MNHN.F.A53758), A2: view of anterior side, left statolith (MNHN.F.A53759). B. *Todaropsis* sp, view of anterior side, right statolith (UBGD 30155 SD (CDN155 in [22]), specimen from the western Mediterranean Sea (area of Banyuls-sur-Mer), mantle length: 70.00 mm, C. Drawing of right statolith of *Martialia hyadesi* Rochebrune and Mabille, 1889 (from [55], modified).

### Studied specimens

MNHN.F.A53758 (Thiverval-Grignon, La Falunière, Fig 8A1), MNHN.F.A53759 (Thiverval-Grignon, La Falunière, Fig 8A2)

### Description

Statoliths typically longer than wide (dorso-ventral sizes are 0.81 mm and 0.82 mm). Dorsal dome small and not well differentiated. Lateral dome seems absent: very small and not distinguishable from the dorsal edge. No groove or any mark in the center of the statolith that may help to distinguish this lateral dome. This imparts a characteristic dorsally broad and roundish shape to the statolith. Ventral rostrum reduced with acuminate ventral termination. Edge between lateral dome and ventral rostrum has a slight inflection point. External part of the wing displays a small to medium ventral indentation a little bit larger than the dorsal one. Main spur more or less marked.

### Comparison

These two statoliths are similar in shape to some Ommastrephidae. They are assigned to that family, with no more precise an assignation. Squids belonging to this family are medium to large (ML from 10 cm to 100 cm), and mostly pelagic. Recent genera from this family include *Illex*, *Todarodes*, *Todaropsis*, *Nototodarus*, *Martialia*, *Ommastrephes*, *Sthenoteuthis*, *Dosidicus*, *Eucleoteuthis*, *Ornithoteuthis*, and *Hyaloteuthis*. The statoliths under review are similar in shape to those of *Todaropsis* sp (Fig 8B), and *Martialia hyadesi* Rochebrune and Mabille, 1889, (see [55,56], and Fig 8C). Although belonging to the Ommastrephidae, the species *Dosidicus lomita* Clarke and Fitch, 1979 from the Late Pliocene has very different statoliths. It is impossible at present to give a more precise taxonomic attribution for these two fossil statoliths.

### Occurrence

The species is known from the Thiverval-Grignon section (see Table 1) from the Middle Lutetian and has an approximate age of 46 to 43 Mya.

## Discussion

### Other cephalopods from the studied outcrops

Five coleoid genera are recorded in the different outcrops studied (Middle Lutetian) [57–59]. The exhaustive list of species that may be found in the Lutetian outcrops of the Paris Basin (11 species occur) is given in S3 Table. In the Thiverval-Grignon section (the best known section), coleoid fossils come mainly from levels 3b to 4 (see S1 Fig). All of them concern the order Sepioida simply because species belonging to this order display an internal shell that may be fossilized (see [5] for an up-to-date review of their evolution). On the contrary no fossils from Teuthida have been discovered yet: they have an internal gladius (not a shell) that is barely fossilized in the studied outcrops. Among the Sepioida discovered, *Beloptera* is rare, and it has not been possible to indicate its exact occurrence level. *Belosaepia* is relatively frequent in levels 5 and 6. *Belosepiella*, a genus whose specimens are smaller than previous genera, is easily found. It is known from more than twenty localities in the Paris Basin of this age. This genus is relatively frequent in levels 3b and 4 which also contain statoliths. The last recorded genus is *Pseudosepia* whose fossils are small and frequently broken.

It is unfortunately impossible to link these fossils to discovered statoliths, mainly because of taphonomic conditions of the studied outcrops: the fossils are mixed together in the sediment. By contrast, some exceptional outcrops display entirely preserved specimens, including soft-parts (e.g. [9]). Within these preservation conditions, we could expect to discover both the shell and associated statoliths. This is absolutely not the case in the studied outcrops. For that reason we have favored a (para)taxonomic identification based on comparison with recent material rather than conjectural assumptions to link macrofossils to the statoliths discovered. Furthermore, this would invariably lead to no Teuthida being identified due to the absence of macrofossils from this group. Using this method, we attribute here several coleoid groups to the Middle Lutetian marine biocenosis: Teuthida (and more precisely specimens from the genus *Loligo* and family Ommastrephidae), and a new Sepioida genus (*Sepia*). The case of? *Sepia pira* sp. nov. is more complex as its taxonomic identification remains uncertain. In a speculative way, we could suggest that instead of being related to *Sepia*, this species might be related to one of the four known genera of macrofossils.

### The fossil record of statoliths: temporal and paleogeographical considerations

**Fig 9:**
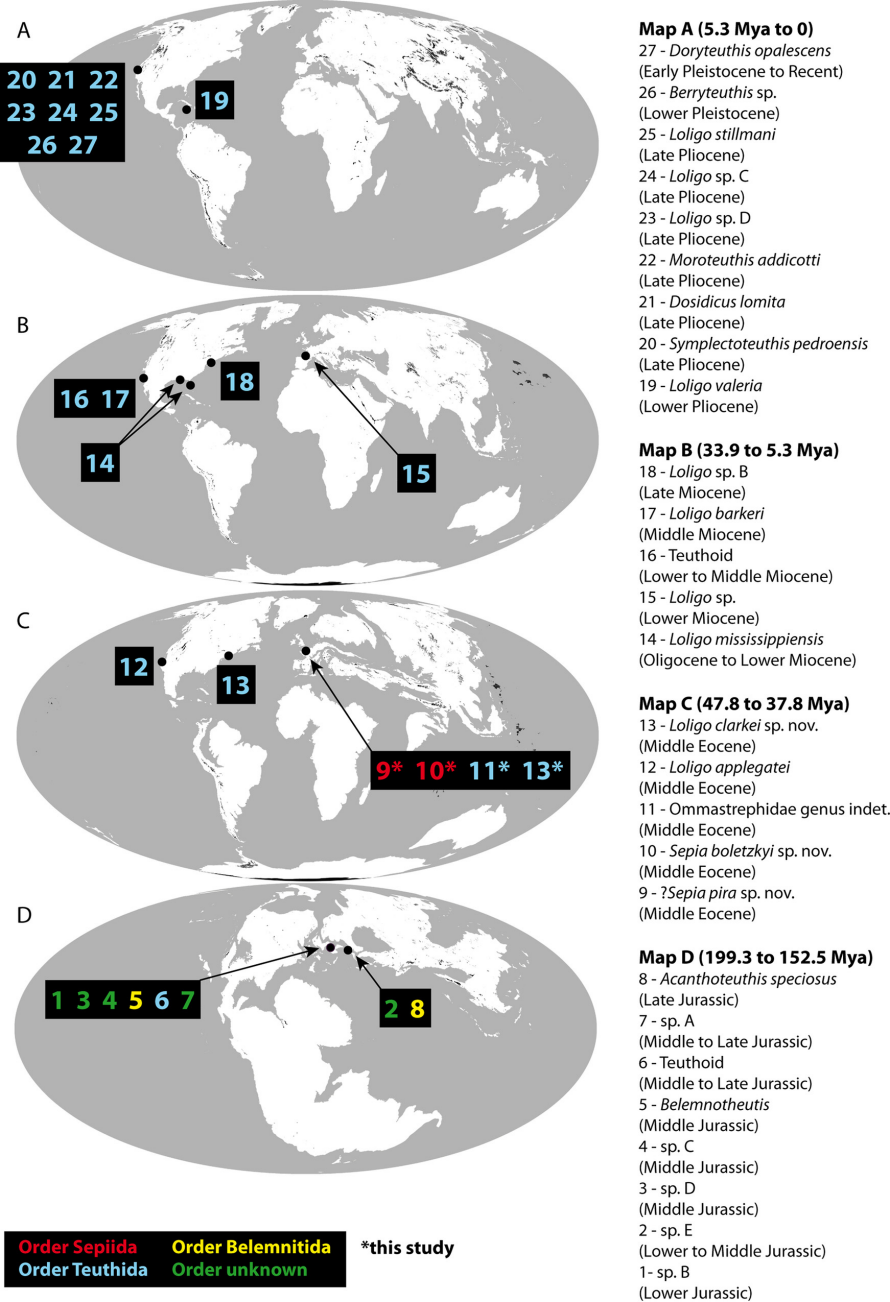
Paleogeographical distributions of the coleoids from their statolith fossil record. Paleogeographical reconstructions modified and redrawn from [60]. Note that for simplification, we choose only one paleogeographical map for several geological ages. Species names and open nomenclature (see Text and S1 Table for further explanations) refer to published data and to the present publication (asterisk). *Loligo clarkei* sp. nov. (label #13) is known from the Paris Basin (this study) and from the east coast of North America under the name *Loligo* sp. A (in [12], see text and S1 Table) *Doryteuthis opalescens* (Berry, 1911) represented here on the map only for fossil specimens. A. Statoliths from Early Pliocene to Early Pleistocene (3 Mya paleogeographical map). B. Statoliths from Oligocene to Late Miocene (20 Mya paleogeographical map). C. Statoliths from Middle Eocene (50 Mya paleogeographical map). D. Statoliths from Early Jurassic to Late Jurassic (170 Mya paleogeographical map).

Thanks to what we consider to be a complete compilation of the literature (S1 Table), and using paleogeographical maps [60], it is now possible to propose a time and space analysis of the statolith fossil record (Fig 9). This should be seen as a first step towards improved knowledge of these fossils.

The earliest recorded statolith is from the Early Jurassic (Sinemurian stage, 199.3 to 190.8 Mya). The highly original shape of this statolith makes its taxonomic identification impossible. Only eight records are known from the entire Jurassic. All are from Southern England, save two from Germany (however, one has never been figured, and the other is not described but only localized in a belemnoid fossil, see S1 Table). These eight records are localized from the Jurassic northwest European Platform. One of them is tentatively referred to as “teuthoid”, although it was impossible to give a more precise identification [16]. Two are referred to the genus *Acanthoteuthis* and *Belemnotheutis* (Order Belemnitida, see [61] for taxonomic considerations), attested by the nearly complete preservation of the organism. However, in these two cases, statoliths are not described, but only localized on the fossil. The others are unidentified. Statolith records from the Jurassic are frequently associated with hooks, even sometimes from a same specimen [62,63]. This is not the case here, and no hook has ever been found in the studied outcrops.

It is surprising to observe that–as far as we know–no statolith has ever been published from the Cretaceous. This may be because micropaleontological samples are generally scarcer than those specifically searched for here, preventing any statolith discoveries. Another limitation would be that the Cretaceous “calcitic sea” poorly preserved aragonitic elements, thus preventing any statolith preservation [64].

By contrast, most of the records are for the Cenozoic. During the Paleogene, statoliths are recorded only in the Middle Eocene (47.8 to 37.8 Mya), including the results of the present paper. Data come from North America (West and East coasts) and from the southern and eastern edges of the Paris Basin. Their taxonomic identification is easier due to their shapes, which resemble recent coleoid statoliths. Interestingly, the new species *Loligo clarkei* n. sp. presented here is also known from the east coast of North America coast at the same age (known as *Loligo* sp. A. [12]). It may be, then, that this species was distributed throughout the North Atlantic. Our study documents for the first time statoliths from this age that can be identified as sepiids, and more particularly as the genus *Sepia*. Fourteen records of fossil statoliths occur in the most recent part of the Cenozoic (33.9 Mya to Quaternary). Different teuthoid genera known today have been recorded (*Loligo*, *Sthenoteuthis*, *Dosidicus*, *Berryteuthis*, *Doryteuthis*), and even one recent species is identified in the fossil record (*Doryteuthis opalescens* (Berry, 1911), see [13]) from an age no more than 2.6 Mya. All records from this recent part of the Cenozoic come from North America (west and east coasts), except one (record 15, Fig 9, and see S1 Table) from the Burdigalian stage (20.4 to 16.0 Mya) of the Aquitanian Basin (France). It is difficult to interpret these time and space patterns more precisely at the present stage of the analysis because of the scarcity of data. Globally, we can only say that all records regardless of age are from the northern hemisphere (Fig 9). The two main coleoid orders are recorded (Sepioida and Teuthida), together with a third, unknown, one. The lack of records during the Cretaceous is certainly a research bias that needs to be corrected in the future.

### Outputs for temporal constraints in molecular phylogenies

As previously stated, the phylogeny of coleoid cephalopods is far from being completely resolved. Beyond pure phylogenetic relationships, another question, relative to the temporal calibration of the phylogeny is still subject to debate. In this context, fossils may contribute directly in phylogenies (when constructing phylogenies based on anatomical characters, and mixing together present and past animals) or indirectly by providing fossil time constraints to estimate divergence times of some clades when calibrating molecular phylogenies. In a recent review of the contribution of molecular data to our understanding of cephalopod evolution [65], the authors conclude that much has been done, but parts remain to be detailed, especially deeper-level relationships. Their review also pointed out the value of discovering, among other things, “new pertinent fossils” to pinpoint divergence times.

The data presented here may be seen as “new pertinent fossils” in this context (see also [66] for very recent findings about fossil coleoid jaws). As pointed out [7], fossilized statoliths provide good evidence for the existence of fossils belonging to the order Teuthoidea. The results presented here further that knowledge a little. *Loligo* was known from several previously published fossil statoliths (see S1 Table). Among them, the genus was already known in the Middle Eocene, as confirmed here. By contrast, but still with a doubt, we suggest here some very new data concerning the family Ommastrephidae: its presence in the Lutetian of the Paris Basin. A minimum age of 3 Mya (Late Pliocene) was used by [7] to calibrate the diversification of this family, based on statolith data [13,16]. Our findings clearly challenge this very recent age, and suggest an age between 46 to 43 Mya to calibrate for the calculation of the diversification age of the family.

Our data also challenge constraints used to find divergence times for the Sepiidae family. Most recent studies [7] assume a minimum date of 43 Mya, based on macrofossils such as *Belosaepia*, to calibrate phylogenies and date the split between the Sepiidae and Spirulidae. The calculated age for that split, using this calibration, ranges from 260 [7] to 150 Mya [67]. These studies also used a minimum of 19 Mya (based on a personal communication of D.T. Donovan), to calibrate phylogenies and find the divergence age of the family Sepiidae.

The new fossil statoliths of *Sepia boletzkyi* n. sp. and? *Sepia pira* n. sp. discovered and described here add two interesting points. The genus *Sepia* is known as early as the Lutetian for an age between 46 to 43 Mya. Using those new findings for the presence of the genus *Sepia* clearly challenges the calibration age used to calculate the diversification age of the family Sepiidae (see above). We recommend using a calibration age of 46 to 43 Myr in future studies, which is very close to the ages used for the Sepidae and Spirulidae split. Whatever this divergence age may be, the presence of the genus *Sepia* at the age of 46 to 43 Myr suggests an explosive diversification (an evolutionary radiation) of the family at that age (at least seen in the fossil record), or even a little earlier (Early Eocene) [5], with the emergence of several genera such as *Sepia*, and *Pseudosepia*, *Beloseapia*, and *Belosepiella* for fossil genera (see S3 Table). New statolith findings, and especially if we strive to find some in the Cretaceous, will certainly make a major contribution to our knowledge of the coleoid cephalopods clade.

## Conclusion

New fossil statoliths described here (*Sepia boletzkyi* sp. nov.,? *Sepia pira* sp. nov., *Loligo clarkei* sp. nov., Ommastrephidae genus indet.) from the Middle Lutetian of the Paris Basin further our knowledge of the coleoid cephalopods clade. They confirm that fossil statoliths are “neglected fossils” and argue in favor of intensifying the search for them, especially in Cretaceous times. The findings described here are mostly congruent with those previously published, both in terms of paleogeographical and temporal distribution. However, we suggest a diversification of the family Ommastrephidae and an emergence of the genus *Sepia* as early as 46 to 43 Mya. This clearly challenges previously published molecular phylogenies calibrated by fossils with a date of 3 Mya and 19 Mya, respectively.

## Supporting Information

S1 Fig The Thiverval-Grignon section (La Falunière, Yvelines, France). The description of the section is modified from [36]. The red arrows indicate the location of the statolith samples. The correlation between the sequential (A6 to A8) and the lithological units is based on [38].
(TIF)

S2 Fig The Lubin-de-la-Haye section (Eure et Loir, France). The description of the section is modified from [32]. The red arrows indicate the location of the statolith samples. The correlation between the sequential (A7 to A8) and the lithological units is based on [38].
(TIF)

S3 Fig The Maulette section (Yvelines, France). The description of the section is modified from [41]. The red arrows indicate the location of the statolith samples. The correlation between the sequential (A6 to A7) and the lithological units is based on J.-P. Gély (written communication).
(TIF)

S4 Fig The Fleury-la-Rivière section (Marne, France). The description of the section is modified from [34]. The red arrows indicate the location of the statolith samples. The correlation between the sequential (A6 to A8) and the lithological units is based on [38].
(TIF)

S1 Table Synthetic presentation of known fossil statoliths from the literature, organized from youngest (top) to oldest (bottom) species. This table uses the formal nomenclatural system together with an open (or informal) nomenclature, reflecting the published literature. In the same way, geological formations and geographical localities follow the published literature. Ages from [42] (at the stage level when information is available). The stars (*) indicate no figuration of fossil statoliths.
(DOC)

S2 Table A. **Size of statoliths for fossil species under study**. Only sufficiently well preserved specimens have been measured, except for the holotype of *Sepia boletzkyi* sp. nov., which is incomplete but measured with a reconstruction of its dorsal dome (see Fig 5A). B. Size of statoliths and mantle length of several recent species for comparison with fossil statolith length. Specimens measured here are those used for morphometric analysis (see [22] and text). All specimens are from the western Mediterranean Sea (area of Banyuls-sur-Mer). All are housed at the University of Burgundy (Dijon, France).
(DOC)

S3 Table Exhaustive list of coleoid cephalopods from the Lutetian of the Paris Basin known from macrofossils (based on [59]). (DOC)
