# Supplementary figures and images for: New Eocene Coleoid (Cephalopoda) Diversity from Statolith Remains: Taxonomic Assignation, Fossil Record Analysis, and New Data for Calibrating Molecular Phylogenies
Source: PLoS One. 2016 May 18;11(5):e0154062. doi: 10.1371/journal.pone.0154062 (PMC4871424; doi:10.1371/journal.pone.0154062)

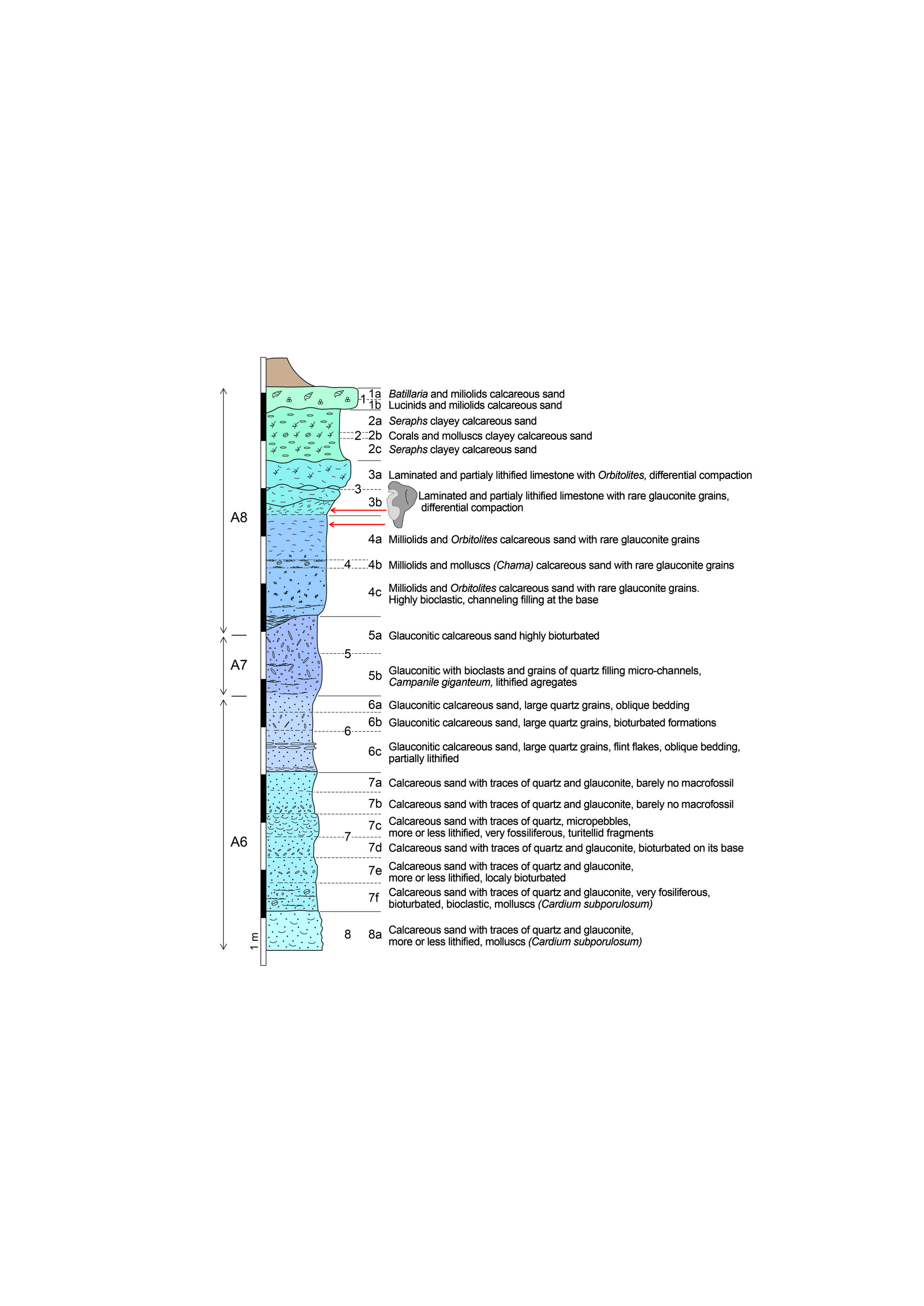

Supplement: S1 Fig — The description of the section is modified from [36]. The red arrows indicate the location of the statolith samples. The correlation between the sequential (A6 to A8) and the lithological units is based on [38]. (TIF) [file pone.0154062.s001.tif]

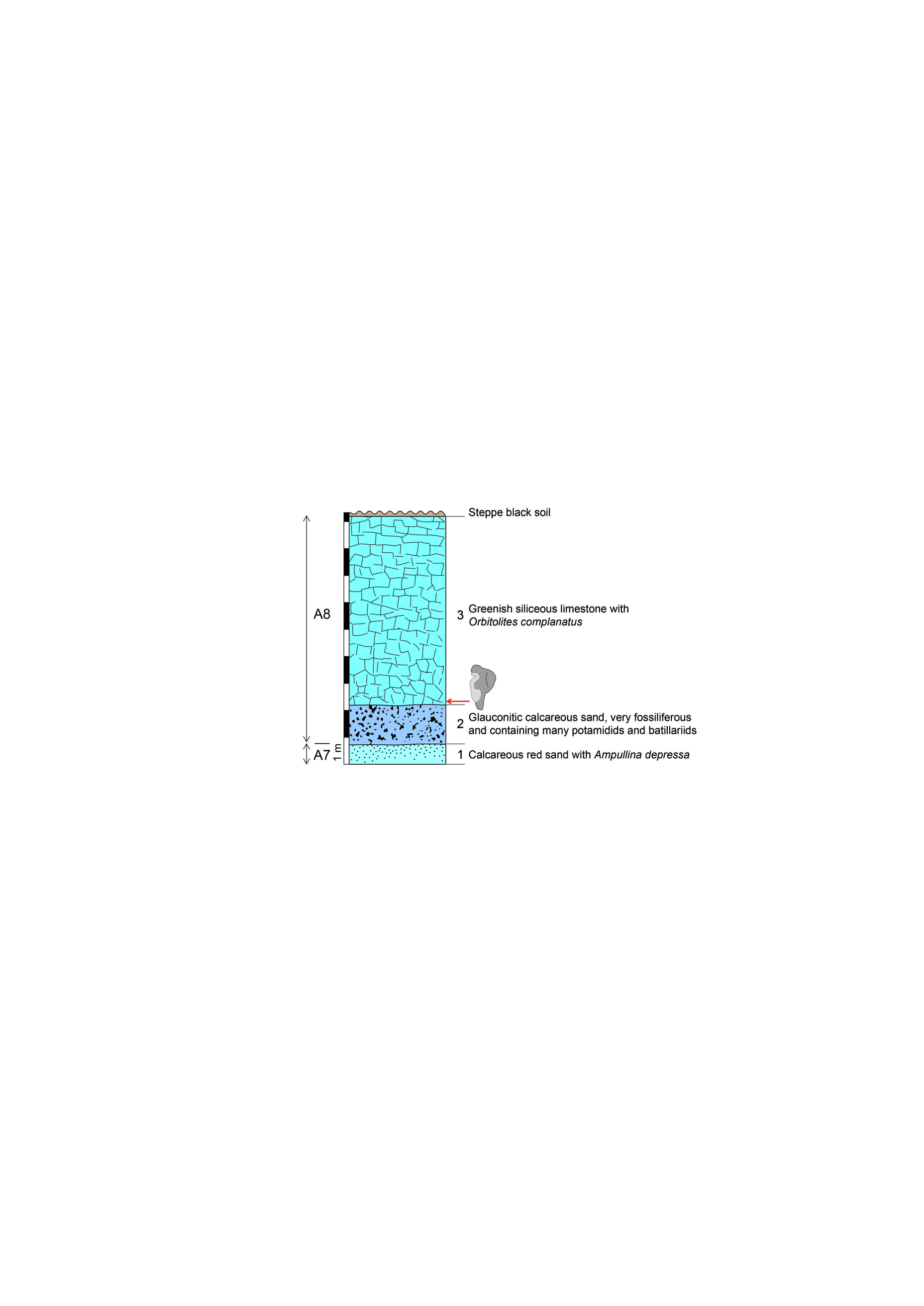

Supplement: S2 Fig — The description of the section is modified from [32]. The red arrows indicate the location of the statolith samples. The correlation between the sequential (A7 to A8) and the lithological units is based on [38]. (TIF) [file pone.0154062.s002.tif]

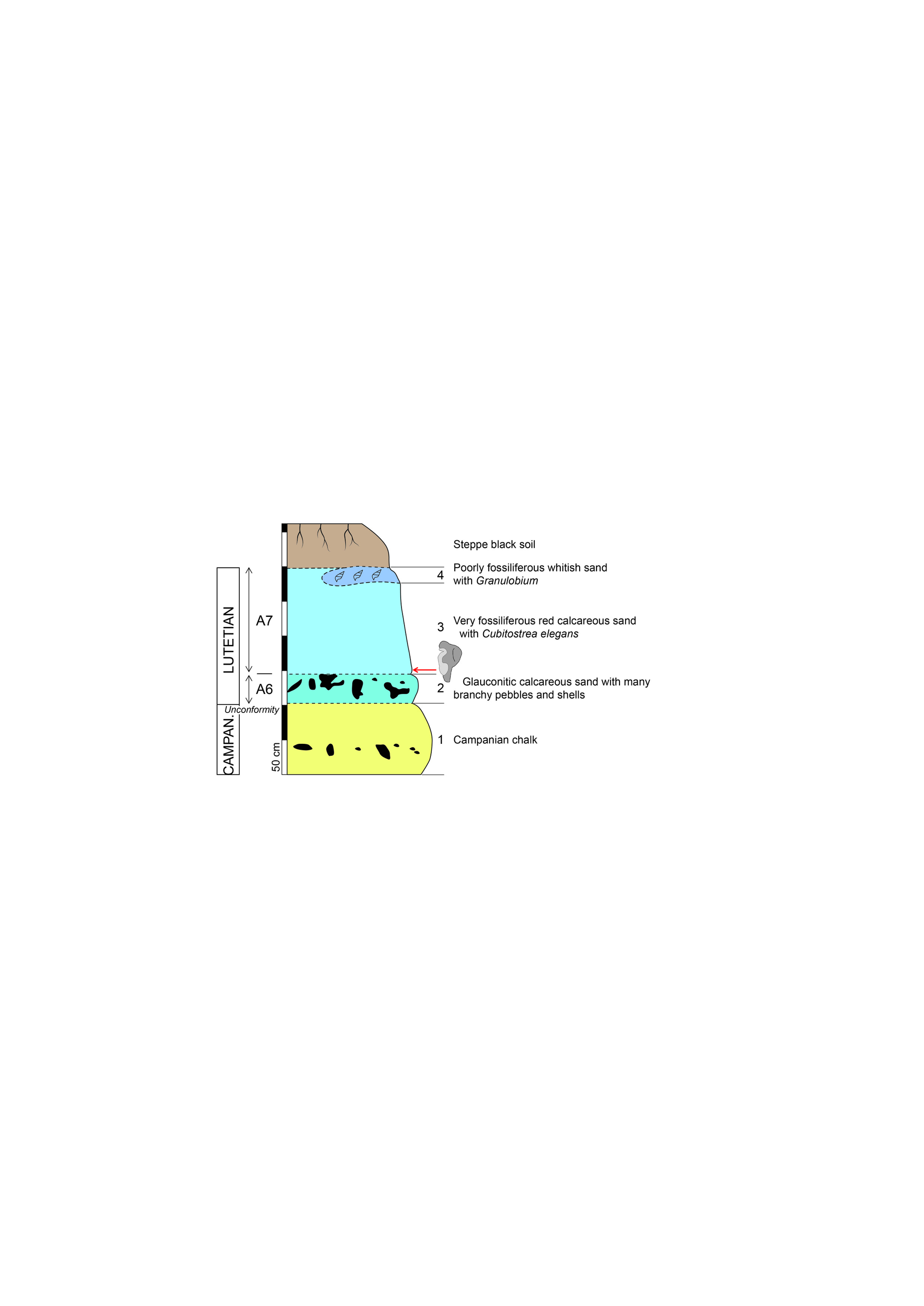

Supplement: S3 Fig — The description of the section is modified from [41]. The red arrows indicate the location of the statolith samples. The correlation between the sequential (A6 to A7) and the lithological units is based on J.-P. Gély (written communication). (TIF) [file pone.0154062.s003.tif]

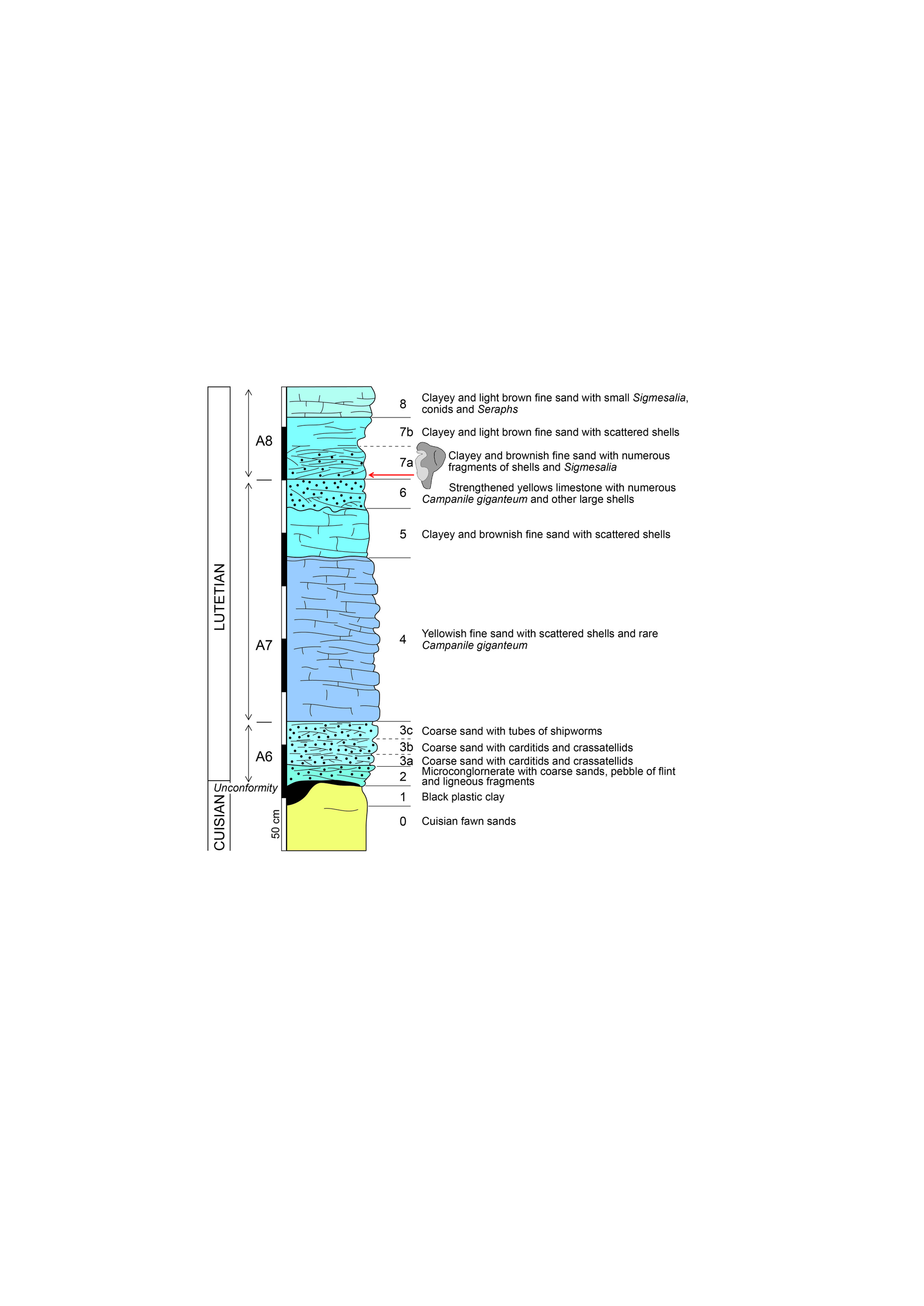

Supplement: S4 Fig — The description of the section is modified from [34]. The red arrows indicate the location of the statolith samples. The correlation between the sequential (A6 to A8) and the lithological units is based on [38]. (TIF) [file pone.0154062.s004.tif]
