# Supplementary material for: New Eocene Coleoid (Cephalopoda) Diversity from Statolith Remains: Taxonomic Assignation, Fossil Record Analysis, and New Data for Calibrating Molecular Phylogenies
Source: PLoS One. 2016 May 18;11(5):e0154062. doi: 10.1371/journal.pone.0154062 (PMC4871424; doi:10.1371/journal.pone.0154062)
Supplement: S1 Table — This table uses the formal nomenclatural system together with an open (or informal) nomenclature, reflecting the published literature. In the same way, geological formations and geographical localities follow the published literature. Ages from [42] (at the stage level when information is available). The stars (*) indicate no figuration of fossil statoliths. (DOC) [file pone.0154062.s005.doc]

**Name System Age (Ma)**

****Doryteuthis opalescens* (Berry, 1911) Early Pleistocene to Recent -2.6 to 0.0**

This Recent species is now classified in the genus *Doryteuthis* (see [1]). [2] (p. 504) mentioned three fossil statoliths of this species (they used the original Linnaean combination: *Loligo opalescens* Berry 1911). However, they do not figure any statolith in their publication. Known from Rincon Hill Road, Santa Barbara (California, USA) for fossils and in inshore waters of the west coast of the USA for recent representatives.

***Berryteuthis* sp. Clarke & Fitch, 1979 Lower Pleistocene -2.6 to -1.8**

First described by [2] (Plate 55, figs 1-20, and Text-fig. 11). Then same specimens refigured by [4] (Fig. 5A-B), and [3] (Fig. 3 bottom). Known from Newport Mesa-Fernando Formation & Lomita Marl formation (California, USA).

***Loligo stillmani* Clarke & Fitch, 1979 Late Pliocene -3.6 to -2.6**

First described by [2] (Plate 54, figs 6-10). Then same specimens refigured by [3] (Fig. 7 bottom). Known from Lomita Marl formation (California, USA).

***Loligo* sp. C Clarke & Fitch, 1979 Late Pliocene -3.6 to -2.6**

First described by [2] (Text-fig. 7I-L). Then same specimens refigured by [3] (Fig. 6I-L). Known from Newport Mesa-Fernando formation (California, USA).

***Loligo* sp. D Clarke & Fitch, 1979 Late Pliocene -3.6 to -2.6**

First described by [2] (Text-fig. 7M-P). Then same specimens refigured by [3] (Fig. 6M-P). Known from Lomita Marl formation (California, USA).

***Moroteuthis addicotti* Clarke & Fitch, 1979 Late Pliocene -3.6 to -2.6**

First described by [2] (Plate 54, figs 16-20). Then same specimens refigured by [3] (Fig. 3 top). Known from Newport Mesa-Fernando formation (California, USA).

***Dosidicus lomita* Clarke & Fitch, 1979 Late Pliocene -3.6 to -2.6**

First described by [2] (Text-fig. 8A-E). Then same specimens refigured by [3] (Fig. 2A-E). Known from Lomita Marl formation (California, USA).

***Sthenoteuthis pedroensis* Clarke & Fitch, 1979 Late Pliocene -3.6 to -2.6**

This species was first described by [2] (Text-fig. 8F-J) in the genus *Symplectoteuthis*. This genus is now considered as a junior synonym of *Sthenoteuthis*. Then same specimens refigured by [3] (Fig. 2F-J). Known from Lomita Marl formation (California, USA).

***Loligo valeria* Clarke & Fitch, 1979 Lower Pliocene -5.3 to -3.6**

First described by [2] (Text-fig. 3K-O). Then same specimens refigured by [3] (Fig. 4K-O), and [5] (Fig. 12K-O). Known from Bowden area (Jamaica).

***Loligo* sp. B Clarke & Fitch, 1979 Late Miocene -11.6 to -5.3**

First described by [2] (Text-fig. 7E-H) as a species from the Late Miocene or possibly “old Pliocene”. Then same specimens refigured by [3] (Fig. 6H-E). Known from Day’s Point (Virginia, USA).

***Loligo barkeri* Clarke & Fitch, 1979 Middle Miocene -16.0 to -11.6**

First described by [2] (Plate 53, figs 1-16, and Plate 54, figs 1-5). Then same specimens refigured by [3] (Fig. 7 top). Known from Baker’s Ranch & Round Mountain Silt areas (California, USA).

**Teuthoid Lower to Middle Miocene -23.0 to -11.6**

First described by [6] as “Miocene teuthoid” (Fig. 1a, 1c) from Olcese Sand (California, USA). This formation is now known from late Lower to early Middle Miocene [7].

***Loligo* sp. Clarke et al 1980b Lower Miocene -20.4 to -16.0**

First described by [8] (Fig. 1) from the Burdigalian stage. Known from Southern Aquitanian Basin, Poyartin and Saubrigues areas (France).

***Loligo mississippiensis* Clarke & Fitch 1979 Oligocene to Lower Miocene -33.9 to -15.97**

First described by [2] (Plate 54, figs 11-15, and Text-fig. 3F-J). Then same specimens refigured by [3] (Fig. 4F-J), and [5] (Fig. 12F-J). Known from Glendon Limestone (Mississippi, USA) and Chipola formation (Florida, USA).

***Loligo applegatei* Clarke & Fitch, 1979 Middle Eocene -47.8 to -41.2**

First described by [2] (Text-fig. 3A-E). Then same specimens refigured by [3] (Fig. 4A-E), and [5] (Fig. 12A-E). Known from Vacaville Shale (California, USA).

***Loligo* sp. A Clarke & Fitch, 1979 Middle Eocene -47.8 to -37.8**

First described by [2] (Text-fig. 7A-D) as a species from the Lutetian. Then same specimens refigured by [3] (Fig. 6A-D). Known from Wallmeyer’s Bluff area (Hanover County, Virginia, USA).

***Acanthoteuthis speciosus* Münster 1839 Late Jurassic -166.1 to -163.5**

A nearly completely preserved specimen described by [9] (Fig. 1) from the Tithonian. Statoliths are not described *sensu stricto*, but may be located in the specimens thanks to fine fossilization. Known from Solnhofen, Germany,

**sp. A Middle to Late Jurassic -166.1 to -152.1**

First described by [5] (Fig.11) as a ‘Jurassic’ species. One specimen figured by [10] (Fig. 1) as “almost identical to ‘Jurassic’ sp. A”. New specimens figured by [11] (Fig. 2A-B) with age precision (Callovian to Kimmeridgian stages). Known from Christian Malford area (Wiltshire, England). Specimens figured by Clarke apparently lost [11].

**Teuthoid Middle to Late Jurassic -166.1 to –157.3**

First described by [3] as “early teuthoid” (Fig. 11) from the Oxford Clay formation, Southern England. This formation is now known to date from the Callovian to Oxfordian stages [12].

***Belemnotheutis* Middle Jurassic -166.1 to -163.5**

First described by [13] (Plates 1, 2) from the Callovian (Athleta zone). Statoliths are not described *sensu stricto*, but may be located in the specimens thanks to fine fossilization. Known from Rixon Gate Quarry (Wiltshire, England).

**sp. C Middle Jurassic -166.1 to -163.5**

First described by [5] (Fig.15 bottom) as a ‘Jurassic’ species. New specimens figured by [11] (Fig. 2E-F) with age precision (Callovian stage). Known from Christian Malford area (Wiltshire, England). Specimens figured by Clarke apparently lost [11].

**sp. D Middle Jurassic -168.3 to -166.1**

First described by [14] (Fig. 3F) as “unidentified statolith, probably new taxon”, then named “sp. D” by [10] (Fig. 2G: refiguration of specimen in [14], Fig. 3F) with age precision (Bathonian stage). Known from Wattonensis Beds, near Langton Herring (Dorset, England).

***sp. E Lower to Middle Jurassic -182.7 to -170.3**

[10] cite this species as belonging to the Toarcian and Aalenian stages, and recorded in SW Germany (“fide Dr. Wolfgang Riegraf”). However, they do not figure any specimen in their publication.

**sp. B Lower Jurassic -199.3 to -190.8**

First described by [5] (Fig. 15 top) as a ‘Jurassic’ species. New specimens figured by [11] (Fig. 2C-D) with age precision (Sinemurian stage). Known from “locality 1” [11] South of Bath (England). Specimens figured by Clarke apparently lost [11].

**References**

1. Vecchione M, Shea E, Bussarawit S, Anderson F, Alexeyev D, Lu CC, Okutani T, Roeleveld M, Chotiyaputta C, Roper C, Jorgensen E, Sukramongkol N. Systematics of Inod-West Pacific loliginids. Phuket mar. biol. Cent. Res. Bull. 2005;66: 23-26.

2. Clarke MR, Fitch JE. Statoliths of Cenozoic teuthoid cephalopods from north America. Palaeontology. 1979;22: 479-511.

3. Clarke MR, Maddock L. Statoliths of fossil coleoid cephalopods. In: Clarke PR, Trueman ER, editors. The Mollusca Vol. 12, Paleontology and Neontology of cephalopods. Academic Press; 1988. pp. 153-168.

4. Clarke MR, Fitch JE, Kristensen T, Kuboderas T, Maddock L. Statoliths of one fossil and four living squids (Gonatidae: Cephalopoda). J Mar Biol Ass UK. 1980;60: 329-347.

5. Clarke MR. Potential of statoliths for interpreting coleoid evolution: a brief review. Berliner Paläbiol. Abh. 2003;3: 37-47.

6. Clarke MR, Fitch JE. First fossil records of cephalopod statoliths. Nature. 1975;257: 380-381.

7. Prothero DR, Sanchez F, Denke LL. Magnetic stratigraphy of the early to middle Miocene Olcese sand and round mountain silt, Kern county, California. New Mexico Museum of Natural History and Science Bulletin. 2008:44; 357-363

8. Clarke MR, Maddock L, Steurbaut E. The first fossil cephalopod statoliths to be described from Europe. Nature. 1980;287: 628-630.

9. Klug C, Schweigert G, Fuchs D, Kruta I, Tischlinger H. Adaptations to squid-style high-speed swimming in Jurassic belemnitids. Biol. Lett. 2016;12: 20150877.

10. Hart MB, De Jonghe A, Rundle AJ, Smart CW. Statoliths: neglected microfossils. J Micropal. 2013;32: 219-220.

11. Hart MB, Clarke MR, De Jonghe A, Price GD, Page KN, Smart CW. Statoliths from the Jurassic succession of south-west England, United Kingdom. Swiss J Palaeontol. 2015. doi 10.1007/s13358-015-0080-3.

12. Wright JK, Cox BM. British Upper Jurassic Stratigraphy. Peterborough: Geological Conservation Review. Joint Nature Conservation Committee; 2001.

13. Wilby PR, Hudson JD, Clements RG, Hollingworth NTJ. Taphonomy and origin of an accumulate of soft-bodied cephalopods in the Oxford Clay Formation (Jurassic, England). Palaeontology. 2004;47: 1159-1180.

14. Hart MB, De Jonghe A, Grimes ST, Metcalfe B, Price GD, Teece, C. Microfaunal analysis of the Wattonensis Beds (Upper Bathonian) of South Dorset. Geoscience in South-West England. 2009;12: 134-139.
