# Supplementary material for: New Eocene Coleoid (Cephalopoda) Diversity from Statolith Remains: Taxonomic Assignation, Fossil Record Analysis, and New Data for Calibrating Molecular Phylogenies
Source: PLoS One. 2016 May 18;11(5):e0154062. doi: 10.1371/journal.pone.0154062 (PMC4871424; doi:10.1371/journal.pone.0154062)
Supplement: S2 Table — Size of statoliths for fossil species under study. Only sufficiently well preserved specimens have been measured, except for the holotype of Sepia boletzkyi sp. nov., which is incomplete but measured with a reconstruction of its dorsal dome (see Fig 5A). B. Size of statoliths and mantle length of several recent species for comparison with fossil statolith length. Specimens measured here are those used for morphometric analysis (see [22] and text). All specimens are from the western Mediterranean Sea (area of Banyuls-sur-Mer). All are housed at the University of Burgundy (Dijon, France). (DOC) [file pone.0154062.s006.doc]

A. Fossil species studied here

| **Specimen number** | **Species** | **Statolith Length (mm)** |
| --- | --- | --- |
| MNHN.F.A53753 | *Sepia boletzkyi* sp. nov. | 0.89 |
| MNHN.F.A53757 | *?Sepia pira* sp. nov. | 0.93 |
| MNHN.F.A53745 | *Loligo clarkei* sp. nov. | 1.05 |
| MNHN.F.A53746 | *Loligo clarkei* sp. nov. | 1.10 |
| MNHN.F.A53749 | *Loligo clarkei* sp. nov. | 1.00 |
| MNHN.F.A53747 | *Loligo clarkei* sp. nov. | 1.05 |
| MNHN.F.A53752 | *Loligo clarkei* sp. nov. | 1.07 |
| MNHN.F.A53758 | Ommastrephidae genus indet. | 0.82 |
| MNHN.F.A53759 | Ommastrephidae genus indet. | 0.81 |

B. Recent species

| **Specimen number** | **Species** | **Sex** | **Maturation** | **Mantle Length (mm)** | **Statolith Length (mm)** |
| --- | --- | --- | --- | --- | --- |
| UBGD 30001 | *Loligo vulgaris* | Female | Immature | 185.0 | 1.7 |
| UBGD 30002 | *Loligo vulgaris* | ? | Immature | 105.0 | 1.4 |
| UBGD 30005 | *Loligo vulgaris* | ? | ? | 69.0 | 1.4 |
| UBGD 30009 | *Loligo vulgaris* | ? | ? | 86.0 | 1.4 |
| UBGD 30010 | *Loligo vulgaris* | ? | ? | 52.0 | 1.1 |
| UBGD 30011 | *Loligo vulgaris* | ? | ? | 69.0 | 1.3 |
| UBGD 30017 | *Loligo vulgaris* | Female | Mature | 160.0 | 1.7 |
| UBGD 30018 | *Loligo vulgaris* | Female | Mature | 205.0 | 1.8 |
| UBGD 30019 | *Loligo vulgaris* | ? | ? | 155.0 | 1.7 |
| UBGD 30021 | *Loligo vulgaris* | ? | ? | 125.0 | 1.5 |
| UBGD 30022 | *Loligo vulgaris* | ? | ? | 146.0 | 1.7 |
| UBGD 30023 | *Loligo vulgaris* | Male | Mature | 158.0 | 1.7 |
| UBGD 30024 | *Loligo vulgaris* | ? | ? | 113.0 | 1.6 |
| UBGD 30025 | *Loligo vulgaris* | ? | ? | 58.0 | 1.2 |
| UBGD 30027 | *Loligo vulgaris* | ? | ? | 165.0 | 1.7 |
| UBGD 30029 | *Loligo vulgaris* | ? | ? | 143.0 | 1.7 |
| UBGD 30030 | *Loligo vulgaris* | ? | ? | 117.0 | 1.4 |
| UBGD 30031 | *Loligo vulgaris* | Female | Mature | 189.0 | 1.8 |
| UBGD 30041 | *Loligo vulgaris* | ? | ? | 44.0 | 1.1 |
| UBGD 30042 | *Loligo vulgaris* | ? | ? | 54.0 | 1.1 |
| UBGD 30043 | *Loligo vulgaris* | ? | ? | 86.0 | 1.4 |
| UBGD 30045 | *Loligo vulgaris* | ? | ? | 70.0 | 1.4 |
| UBGD 30062 | *Loligo vulgaris* | ? | ? | 52.0 | 1.1 |
| UBGD 30077 | *Loligo vulgaris* | ? | ? | 137.0 | 1.6 |
| UBGD 30078 | *Loligo vulgaris* | Female | Mature | 179.0 | 1.7 |
| UBGD 30087 | *Loligo vulgaris* | ? | ? | 47.0 | 1.0 |
| UBGD 30088 | *Loligo vulgaris* | ? | ? | 42.0 | 1.1 |
| UBGD 30089 | *Loligo vulgaris* | ? | ? | 46.0 | 1.1 |
| UBGD 30101 | *Loligo vulgaris* | Male | Mature | 182.0 | 1.7 |
| UBGD 30104 | *Loligo vulgaris* | Male | Mature | 235.0 | 1.8 |
| UBGD 30105 | *Loligo vulgaris* | Female | Mature | 195.0 | 1.9 |
| UBGD 30110 | *Loligo vulgaris* | Female | Immature | 198.0 | 1.7 |
| UBGD 30111 | *Loligo vulgaris* | Female | Mature | 192.0 | 1.8 |
| UBGD 30008 | *Sepia elegans* | Male | Mature | 39.7 | 1.3 |
| UBGD 30051 | *Sepia elegans* | Female | Mature | 42.0 | 1.3 |
| UBGD 30052 | *Sepia elegans* | Female | Mature | 47.9 | 1.3 |
| UBGD 30053 | *Sepia elegans* | Female | Mature | 40.5 | 1.2 |
| UBGD 30054 | *Sepia elegans* | Female | Mature | 46.8 | 1.3 |
| UBGD 30055 | *Sepia elegans* | Female | Mature | 40.9 | 1.2 |
| UBGD 30056 | *Sepia elegans* | Male | Mature | 42.2 | 1.3 |
| UBGD 30090 | *Sepia elegans* | Female | Immature | 37.6 | 1.2 |
| UBGD 30091 | *Sepia elegans* | Female | Immature | 37.7 | 1.1 |
| UBGD 30092 | *Sepia elegans* | Female | Immature | 27.7 | 1.1 |
| UBGD 30093 | *Sepia elegans* | Male | Mature | 42.1 | 1.3 |
| UBGD 30044 | *Sepia officinalis* | Male | Immature | 67.7 | 1.5 |
| UBGD 30046 | *Sepia officinalis* | ? | ? | 70.6 | 1.6 |
| UBGD 30047 | *Sepia officinalis* | ? | ? | 84.0 | 1.6 |
| UBGD 30048 | *Sepia officinalis* | Female | Mature | 169.8 | 2.0 |
| UBGD 30050 | *Sepia officinalis* | ? | ? | 143.8 | 1.9 |
| UBGD 30134 | *Sepia officinalis* | ? | ? | 190.7 | 2.0 |
| UBGD 30112 | *Sepia officinalis* | ? | ? | 53.0 | 1.2 |
| UBGD 30113 | *Sepia officinalis* | ? | ? | 47.5 | 1.2 |
| UBGD 30114 | *Sepia officinalis* | ? | ? | 59.1 | 1.3 |
| UBGD 30116 | *Sepia officinalis* | ? | ? | 56.7 | 1.2 |
| UBGD 30117 | *Sepia officinalis* | ? | ? | 52.3 | 1.2 |
| UBGD 30119 | *Sepia orbignyana* | Female | Mature | 73.7 | 1.8 |
| UBGD 30120 | *Sepia orbignyana* | Female | Mature | 72.9 | 1.7 |
| UBGD 30121 | *Sepia orbignyana* | Female | Immature | 66.7 | 1.8 |
| UBGD 30122 | *Sepia orbignyana* | Male | Mature | 70.3 | 1.6 |
| UBGD 30123 | *Sepia orbignyana* | Male | Mature | 55.8 | 1.5 |
| UBGD 30124 | *Sepia orbignyana* | Male | Mature | 65.2 | 1.7 |
| UBGD 30126 | *Sepia orbignyana* | Male | Mature | 49.3 | 1.5 |
| UBGD 30127 | *Sepia orbignyana* | Male | Mature | 58.6 | 1.6 |
| UBGD 30128 | *Sepia orbignyana* | Male | Mature | 67.6 | 1.7 |
| UBGD 30129 | *Sepia orbignyana* | Male | Mature | 60.2 | 1.7 |
| UBGD 30130 | *Sepia orbignyana* | Male | Mature | 72.0 | 1.6 |
| UBGD 30154 | *Todaropsis sp* | ? | ? | 77.0 | 0.9 |
