# Supplementary material for: New Eocene Coleoid (Cephalopoda) Diversity from Statolith Remains: Taxonomic Assignation, Fossil Record Analysis, and New Data for Calibrating Molecular Phylogenies
Source: PLoS One. 2016 May 18;11(5):e0154062. doi: 10.1371/journal.pone.0154062 (PMC4871424; doi:10.1371/journal.pone.0154062)
Supplement: S3 Table — (DOC) [file pone.0154062.s007.doc]

Class Cephalopoda Cuvier, 1797

Subclass Coleoidea Bather, 1888

Superorder Decabrachia Haeckel, 1866

Order Sepiida Zittel, 1895

Family Sepiidae Keferstein, 1866

*Pseudosepia vera* (Deshayes, 1865)

Family Belosaepiidae Dixon, 1850

*Belosaepia blainvillii* (Deshayes, 1835)

*Belosaepia brevispina* Sowerby in Dixon, 1850

*Belosaepia oweni* Sowerby in Dixon, 1850

*Belosaepia sepioidea* (de Blainville, 1825)

*Stenosepia compressa* (de Blainville, 1827)

Family Belosepiellidae Naef, 1921

*Belosepiella cossmanni* de Alessandri, 1905

*Belosepiella parisiensis* de Alessandri, 1905

Order Spirulida Stolley, 1919

Family Belopteridae Owen, 1856

*Beloptera (s.str.) belemnoidea* de Blainville, 1825

*Beloptera (s.str.) curta* Cossmann, 1896

*Beloptera (s.str.) edwardsi* Deshayes, 1865
